# Synthesis, characterization, *molecular docking* and in vitro anti-arthritic activity of some novel spiro [1,3,4] thiadiazole derivatives based on thioxoacetamides

**DOI:** 10.1186/s13065-026-01745-w

**Published:** 2026-03-08

**Authors:** Ahmed M. El-Saghier, Asmaa Abdul-Baset, Omar M. El-Hady, Aly Abdou, Amany M. Hamed, Asmaa M. Kadry

**Affiliations:** Chemistry Department, Faculty of Science, Sohag University, Sohag, Egypt

**Keywords:** Anti-arthritic activity, Thioxoacetamide, Spirothiadiazoles, Molecular docking and COX-2 inhibition

## Abstract

A new series of spiro[1,3,4]thiadiazole derivatives based on thioxoacetamides were synthesized, characterized, and evaluated for anti-arthritic potential. The structures of all compounds (**2a–12a**) were confirmed using IR, NMR, and elemental analysis. In vitro anti-arthritic activity was assessed via protein denaturation inhibition and RBC membrane stabilization assays. Compounds **9a**, **10a**, **6a**, **6b**, **6c**, and **6d** showed superior activity compared to the standard drug indomethacin, with **9a** being the most potent (IC₅₀ = 29.6 µg/mL for protein denaturation). Molecular docking against COX-2 (PDB: 5IKT) revealed strong binding affinities, especially for **9a** (− 8.59 kcal/mol), **10a** (− 8.45 kcal/mol), and **6a** (− 8.05 kcal/mol). DFT studies indicated favorable electronic properties comparable to indomethacin. These results highlight the promising anti-arthritic potential of the synthesized spirothiadiazole derivatives.

**Graphical Abstract:**

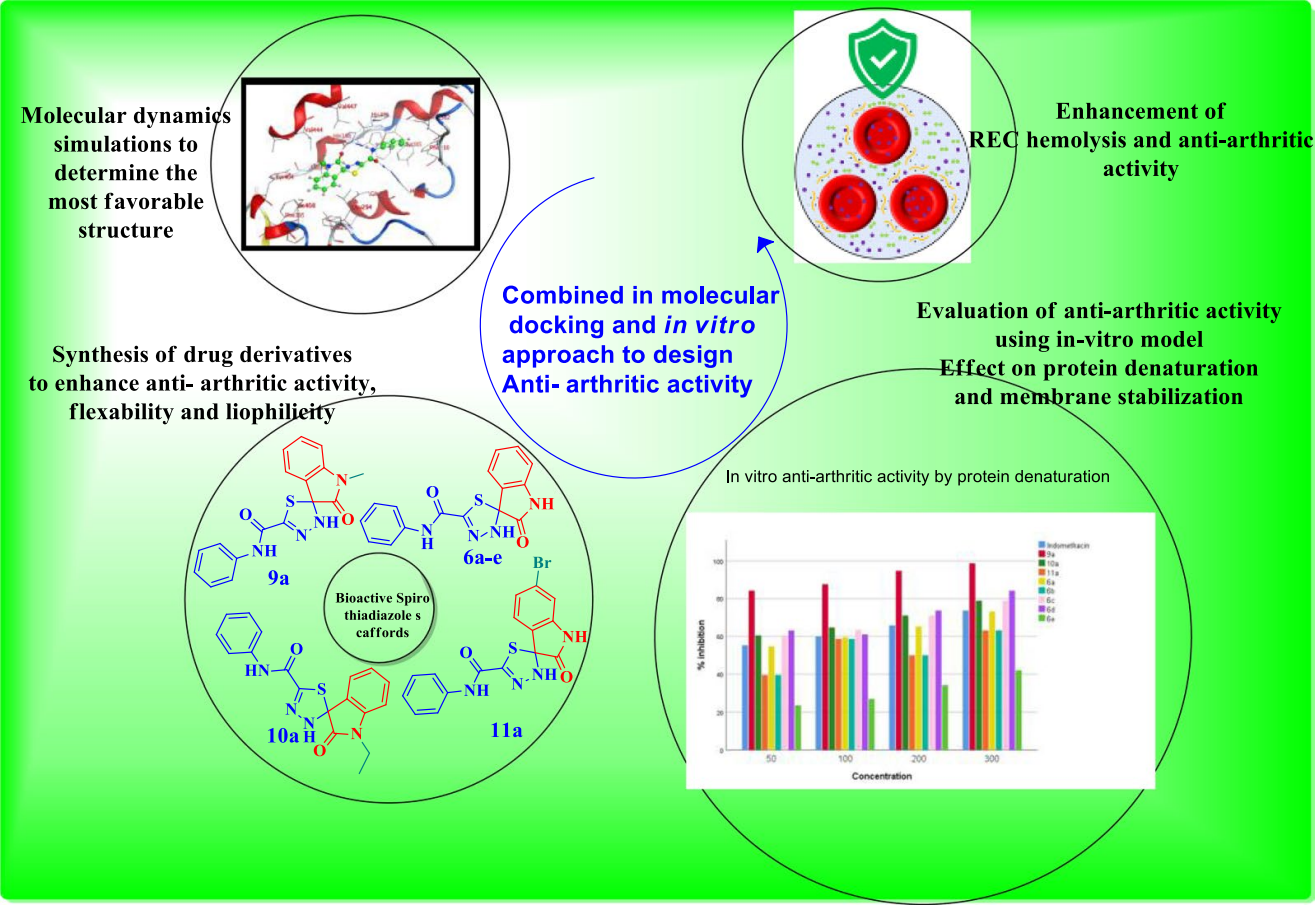

**Supplementary Information:**

The online version contains supplementary material available at 10.1186/s13065-026-01745-w.

## Introduction

Arthritis is a prevalent inflammatory joint disorder affecting millions worldwide, with current treatments like NSAIDs often causing severe side effects such as gastrointestinal bleeding and renal toxicity. Millions of people experience musculoskeletal abnormalities and arthritis globally, which severely restricts their ability to go about their daily lives [1, 3]. It can cause a range of symptoms in affected individuals, from mild ones like mild discomfort and swelling to severe ones like complete or partial joint immobility, muscle atrophy, and contractures, according to Chan and Wu [2]. Additionally, the above-mentioned therapeutic options have the potential to result in fatal outcomes such as severe liver damage, gastrointestinal hemorrhage [4], hospitalization, and death [5]. This underscores the urgent need for safer and more effective anti-arthritic agents.

**Fig. 1:**
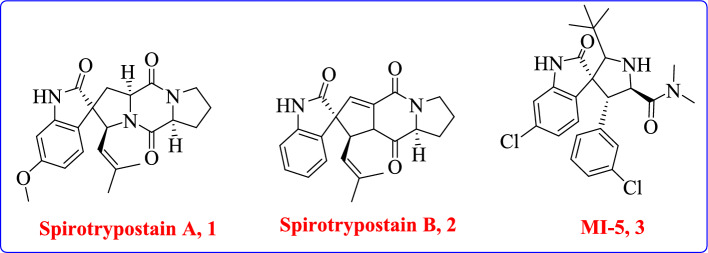
Representative natural and synthetic spiro[pyrrolidine-3,3-oxindole] bioactive compounds

Spiro compounds and 1,3,4-thiadiazole derivatives are recognized for their broad pharmacological profiles [6, 7], including anti-inflammatory, antimicrobial, and anticancer activities. Notably, spiro-thioxanthene derivatives have demonstrated promising anti-inflammatory effects, suggesting the potential of spiro-thiadiazole hybrids as novel therapeutic scaffolds [8–17]. For example, spirooxindole MI-5, 3 demonstrated a novel type of outlawing of the p53-MDM2 protein–protein interaction, which is climactical for regulating the tumor crackdown efficaciousness of the p53-proteins. Spirotryptostatin**A** and **B**, **1** and **2** also blocks tubulin polymerization and involves cell cycle inhibition of tumor cells at G2/M stage. (Fig. 1) [11, 18–20].

Building on our previous work in S,N-heterocycle synthesis [21–24], this study aims to design, synthesize, and evaluate a new series of spiro[1,3,4]thiadiazole derivatives based on thioxoacetamide precursors. We report a facile, high-yield synthetic route and comprehensively assess the anti-arthritic potential of the synthesized compounds using in vitro models (protein denaturation and membrane stabilization), molecular docking against COX-2 (PDB: 5IKT), and DFT calculations to elucidate structure–activity relationships and drug-likeness.

## Methodology

### General information

Thin layer chromatography (TLC) was employed to monitor all reactions using precoated plates of silica gel G/UV-254 with a 0.25 mm thickness (Merck 60F254) and UV light (254 nm/365 nm) for visibility. All melting points were recorded using the uncorrected Kofeler melting point instrument. IR spectra were analyzed using KBr pellets on an FT-IR spectrophotometer. ^1^H NMR and ^13^C NMR (DMSO-d6) spectra were recorded at 400 M Hz and 100 MHz, respectively, at Sohag University. For ^1^H NMR, the following data are given: chemical shift, multiplicity (s = singlet, d = doublet, t = triplet, and m = multiplet), and integration. Parts per million (ppm) of chemical shifts (δ) were reported using tetramethylsilane (TMS) as an internal standard (= 0 ppm) for ^13^C NMR. TMS (= 0 ppm) or DMSO (= 39.51 ppm) were used as internal standards. The elemental analyses were provided by a Perkin-Elmer CHN analyzer model.

### Synthesis of organic compound

#### General synthesis of compounds 2a-7a-d

A mixture of thioxoacetamides **(2a-e)** (1.0 mmol, 1.0 equiv), the corresponding carbonyl compound (mono or di ketone) such as cyclopentanone, cyclohexanone, 2-methylcyclohexanone, benzophenone,isatin or acenaphthylene-1,2-dione (1.0 mmol, 1.0 equiv) was dissolved in absolute ethanol (10 mL) and refluxed for 2 h (monitored with TLC). After cooling, the formed precipitate was then filtered and recrystallized from ethanol.

##### *N*-phenyl-4-thia-1,2-diazaspiro[4.4]non-2-ene-3-carboxamide (2a)

White crystals, yield 84%, mp. 145–147 ºC FT-IR (ATR) *ν*_max_: 3441, 3241 (2NH), 1673 (C = O); ^1^H NMR: *δ* 10.32 (s, H, NH_amidic_, exchangeable by D_2_O), 8.93 (s, H, NH_cyclic_, exchangeable by D_2_O), 7.89- 7.21 (m, 5H, ArH), 2.06–1.63 ppm (m, 8H, 4CH_2_cyclopentanone); ^13^C NMR: *δ* 158.29 (C = O), 151.75 (C, Thiadiazole), 142.54, 134.30, 125.21, 120.74 (Arom.), 86.46 (spiro), 30.60, 25.87 ppm cyclopentanone. Dept -135 NMR; 127.74, 122.73, 119.84 (Arom.), 26.17, 24.66 ppm. Anal. Calcd. for C_13_H_15_N_3_OS (261.34): C, 59.74; H, 5.79; N, 16.08; S,12.27% Found: C, 59.72; H, 5.83; N, 16.17; S, 12.38%.

##### *N*-phenyl-4-thia-1,2-diazaspiro[4.5]dec-2-ene-3-carboxamide(3a)

White crystals, yield 88%, mp. 146–148 ºC FT-IR (ATR) *ν*_max_: 3371, 3259 (2NH), 1667 (C = O); ^1^H NMR: *δ* 10.05 (s, H, NH_amidic_, exchangeable by D_2_O), 8.66 (s, H, NH_cyclohexanone_, exchangeable by D_2_O), 7.72- 7.05 (m, 5H, ArH), 2.09–1.19 ppm (m, 10 H, 5CH_2_cyclohexanone); ^13^C NMR: *δ* 158.91 (C = O), 138.62 (C, Thiadiazole), 138.40, 129.03, 124.09, 120.61 (Arom.), 87.68 (spiro), 30.63, 24.98, 24.27 ppm (Cyclohexanon). Dept -135 NMR; 128.72, 123.97, 120.48 (Arom.), 39.45, 24.97, 24.26 ppm. Anal.Calcd. for C_14_H_17_N_3_OS (275.37): C, 61.06; H, 6.22; N, 15.26; S, 11.64% Found: C, 61.19; H, 6.43; N, 15.10; S, 11.56%.

##### 6-Methyl-*N*-phenyl-4-thia-1,2-diazaspiro[4.5]dec-2-ene-3-carboxamide (4a)

White crystals, yield 81%, mp. 155–157 ºC FT-IR (ATR) *ν*_max_: 3344, 3269 (2NH), 1680 (C = O_amidic_); ^1^H NMR: *δ*10.36 (s, H, NH_amidic_, exchangeable by D_2_O), 8.77 (s, H, NH_thiadiazole_, exchangeable by D_2_O), 7.63- 6.98 (m, 5H, ArH), 2.22–1.00 ( m, 9H, 4CH_2_ + CH_aliphatic_), 0.98 ppm (CH_3_);^13^C NMR: *δ* 158.79 (C = O), 139.71 (C, Thiadiazole), 138.48, 129.44, 125.63, 122.52 (Arom.), 79.14 (spiro), 37.61, 29.90, 25.86, 24.84, 24.27 (2-methylcyclohexanon), 13.20 ppm (CH_3_). Anal. Calcd. for C_15_H_19_N_3_OS (289.40): C, 62.25; H, 6.62; N, 14.52; S,11.08% Found: C, 62.53; H, 6.43; N, 14.31; S, 11.22%.

##### *N*,5,5-triphenyl-4,5-dihydro-1,3,4-thiadiazole-2-carboxamide (5a)

Yellow crystals, yield 66%, mp. 274–276 ºC FT-IR (ATR) *ν*_max_: 3454, 3343 (2NH), 1653 (C = O_amidic_); ^1^H NMR: *δ* 11.21 (s, H, NH_amidic_, exchangeable by D_2_O), 10.67 (s, H, NH_hydrazide_, exchangeable by D_2_O), 8.16- 6.66 ppm (m, 15H, ArH); ^13^C NMR: *δ* 166.80 (C = O), 156.23 (C, Thiadiazole), 151.55, 142.87, 139.80, 137.93, 135.37, 131.89, 129.26, 125.46, 121.57 (Arom.), 72.58 ppm (quaternary carbon atom). Anal. Calcd. for C_21_H_17_N_3_OS (359.44): C, 70.17; H, 4.77; N, 11.69; S, 8.92% Found: C, 70.39; H, 4.71; N, 11.65; S, 8.57%.

##### 2-Oxo-*N*-phenyl-3'H-spiro[indoline-3,2'-[1,3,4]thiadiazole]-5'-carboxamide (6a)

Orange crystals, yield 95%, mp. 263–265 ºC FT-IR (ATR) *ν*_max_: 3438, 3344, 3322 (3NH), 1722 (C = O_cyclic_), 1667 (C = O_amidic_); ^1^H NMR: *δ* 10.52, 10.26, 9.51(s, 3H, 3NH, exchangeable by D_2_O, 7.75- 6.87 ppm (m, 9H, ArH); ^13^C NMR: *δ* 175.75 (C = O_cyclic_), 158.07 (C = O_amidic_), 138.67, 138.11 (2C, Thiadiazole), 131.42, 129.88, 129.08, 129.32, 124.37, 123.28, 120.85, 110.73 (Arom.), 80.57 ppm (spiro). Anal. Calcd. for C_16_H_12_N_4_O_2_S (324.36): C, 59.25; H, 3.73; N, 17.27; S, 9.89% Found C, 58.99; H, 3.95; N, 17.26; S, 9.81%.

##### 2-Oxo-*N*-(o-tolyl)-3'H-spiro[indoline-3,2'-[1,3,4]thiadiazole]-5'-carboxamide (6b)

Orange crystals, yield 77%, mp. 259–261 ºC FT-IR (ATR) *ν*_max_: 3415, 3250,,3190 (3NH), 1700 (C = O_cyclic_), 1672 (C = O_amidic_); ^1^H NMR: *δ* 10.59, 9.78, 9.50(s, 3H, 3NH, exchangeable by D_2_O), 7.56- 6.88 (m, 8H, ArH), 2.23 ppm (s, 3H, CH_3_); ^13^C NMR: *δ* 175.80 (C = O_cyclic_), 158.07 (C = O_amidic_), 141.64, 137.90 (2C, Thiadiazole), 135.94, 133.30, 131.40, 130.79, 129.47, 126.53, 126.35, 126.13, 123.29, 110.43 (Arom.), 80.53 (spiro), 18.18 ppm (CH_3_). Anal. Calcd. for C_17_H_14_N_4_O_2_S (338.38): C, 60.34; H, 4.17; N, 16.56; S, 9.98% Found: C, 60.41; H, 4.35; N, 16.33; S, 9.75%.

##### *N*-(4-Methoxyphenyl)-2-oxo-3'H-spiro[indoline-3,2'

**[1,3,4]thiadiazole]-5'-carboxamide (6c)**

Orange crystals, yield 71%, mp. 255–257 ºC FT-IR (ATR) *ν*_max_: 3437, 3219, 3122 (3NH), 1700 (C = O_cyclic_), 1677 (C = O_amidic_); ^1^H NMR: *δ* 10.73, 10.36, 9.55 (s, 3H, 3NH, exchangeable by D_2_O), 7.79- 6.95 ppm (m, 8H, ArH), 3.88 ppm (OCH_3_); ^13^C NMR: *δ* 176.29 (C = O_cyclic_), 159.86 (C = O_amidic_), 138.96, 136.91 (2C, Thiadiazole), 133.67, 131.17, 129.44, 128.41, 125.47, 123.11, 120.85, 115.49 (Arom.), 81.23 (spiro), 56.14 ppm (OCH_3_). Anal. Calcd. for C_17_H_14_N_4_O_3_S (354.38): C, 57.62; H, 4.98; N, 15.81; S,9.05% Found: C, 57.74; H, 4.01; N, 15.70; S, 9.03%.

##### *N*-(4-Nitrophenyl)-2-oxo-3'H-spiro[indoline-3,2'-[1,3,4]-thiadiazole]-5'-carboxamide (6d)

Orange crystals, yield 70%, mp. 270–272 ºC FT-IR (ATR) *ν*_max_: 3438, 3344, 3322 (3NH), 1722 (C = O_cyclic_), 1667 (C = O_amidic_), 1330–1535 (NO_2_); ^1^H NMR: *δ* 10.97, 10.62, 9.79 (s, 3H, 3NH, exchangeable by D_2_O), 8.26- 6.87 ppm (m, 8H, ArH); ^13^C NMR: *δ* 175.68 (C = O_cyclic_), 158.66 (C = O_amidic_), 143.13, 141.67 (2C, Thiadiazole), 137.07, 131.25, 129.23, 128.14, 126.38, 124.95, 123.36, 120.72, 110.81 (Arom.), 80.83 ppm (spiro). Anal. Calcd. for C_16_H_11_N_5_O_4_S (369.35): C, 52.05; H, 3.00; N, 18.96; S, 8.68% Found: C, 52.32; H, 3.07; N, 18.83; S, 8.37%.

##### *N*-(4-Chlorophenyl)-2-oxo-3'H-spiro[indoline-3,2'-[1,3,4]-thiadiazole]-5'-carboxamide (6e)

Orange crystals, yield 66%, mp. 270–26 ºC FT-IR (ATR) *ν*_max_: 3355, 3251, 3176 (3NH), 1724 (C = O_cyclic_), 16,675 (C = O_amidic_); ^1^H NMR: *δ* 10.61, 9.73, 9.64 (s, 3H, 3NH, exchangeable by D_2_O), 7.79- 6.86 ppm (m, 8H, ArH); ^13^C NMR: *δ* 175.38 (C = O_cyclic_), 157.74 (C = O_amidic_), 141.65, 136.97 (2C, Thiadiazole), 134.33, 131.42, 131.22, 129.88, 129.38, 128.21, 127.24, 126.41, 125.83, 123.26, 110.62 (Arom.), 81.05 ppm (spiro). Anal. Calcd. for C_16_H_11_ClN_4_O_2_S (358.80): C, 53.56; H, 3.09; Cl, 9.88; N, 15.61; S,8.94% Found: C, 53.68; H, 3.21; Cl, 9.76; N, 15.52; S, 8.92%.

##### 2-Oxo-*N*-phenyl-2H,3'H-spiro[acenaphthylene-1,2'-[1, 3, 4]-thiadiazole]-5'-carboxamide (7a)

Orange crystals, yield 94%, mp. 278–280 ºC FT-IR (ATR) *ν*_max_: 3316, 3253 (2NH), 1724 (C = O_cyclic_), 1665 (C = O_amidic_); ^1^H NMR: *δ* 10.42, 9.64 (s, 2H, 2NH, exchangeable by D_2_O, 8.42- 7.10 ppm (m, 11H, ArH); ^13^C NMR: *δ* 198.73 (C = O_cyclic_), 158.31 (C = O_amidic_), 140.49 (C, Thiadiazole), 138.65, 138.11, 137.14, 132.99, 130.10, 129.89, 129.13, 128.87, 127.06, 124.42, 124.20, 123.15, 120.83 (Arom.), 83.96 ppm (spiro). Anal. Calcd. for C_20_H_13_N_3_O_2_S (359.40): C, 66.84; H, 3.65; N, 11.69; S, 8.92% Found: C, 66.75; H, 3.62; N, 11.70; S, 8.94%.

##### 2-Oxo-*N*-(o-tolyl)-2H,3'H-spiro[acenaphthylene-1,2'-[1, 3, 4]-thiadiazole]-5'-carboxamide (7b)

Orange crystals, yield 77%, mp. 280-282ºC FT-IR ºC FT-IR (ATR) *ν*_max_: 3283, 3258 (2NH), 1717 (C = O_cyclic_) 1684 (C = O_amidic_); ^1^H NMR: *δ* 10.37, 9.21 (s, 2H, 2NH, exchangeable by D_2_O, 8.41- 7.08 ppm (m, 10H, ArH); ^13^C NMR: *δ* 195.62 (C = O_cyclic_), 158.22 (C = O_amidic_), 139.00 (C, Thiadiazole), 138.52, 137.94, 137.06, 131.74, 130.73, 129.77, 128.87, 128.59, 126.99, 125.75, 124.65, 123.88, 122.13, 121.29, 121.08 (Arom.), 82.54 (spiro), 19.26 ppm (CH_3_). Anal. Calcd. for C_21_H_15_N_3_O_2_S (373.43): C, 67.54; H, 4.05; N, 11.25; S, 8.59% Found: C, 67.51; H, 4.09; N, 11.28; S, 8.58%.

##### *N*-(4-Methoxyphenyl)-2-oxo-2H,3'H-spiro[acenaphthylene-1,2'-[1,3,4]thiadiazole]-5'-carboxamide (7c)

Orange crystals, yield 81%, mp. 272–274 ºC FT-IR (ATR) *ν*_max_: 3362, 3252 (2NH), 1714 (C = O_cyclic_)1681 (C = O_amidic_); ^1^H NMR: *δ* 10.60, 9.73 (s, 2H, 2NH, exchangeable by D_2_O), 8.41- 7.17 (m, 10H, ArH), ppm; ^13^C NMR: *δ* 198.86 (C = O_cyclic_), 159.72 (C = O_amidic_), 140.79 (C, Thiadiazole), 138.94, 138.53, 138.37, 133.82, 132.15, 130.11, 129.70, 128.67, 128.49, 125.73, 124.28, 123.87, 121.71 (Arom.), 83.25 (spiro), 55.51 ppm (OCH_3_). Anal. Calcd. for C_21_H_15_N_3_O_3_S (389.43): C, 64.77; H, 3.88; N, 10.79; S, 8.23% Found: C, 64.79; H, 3.86; N, 10.82; S, 8.21%.

##### *N*-(4-Nitrophenyl)-2-oxo-2H,3'H-spiro[acenaphthylene-1,2'-[1,3,4]thiadiazole]-5'-carboxamide(7d)

Orange crystals, yield 67%, mp. 255–257 ºC FT-IR (ATR) *ν*_max_: 3246, 3182 (2NH), 1730 (C = O_cyclic_), 1679 (C = O_amidic_) 1344–1524 (NO_2_); ^1^H NMR: *δ* 10.52, 9.73 (s, 2H, 2NH, exchangeable by D_2_O, 8.47- 7.60 ppm (m, 10H, ArH); ^13^C NMR: *δ* 199.55 (-C = O_cyclic_), 159.11 (C = O_amidic_), 140.86 (C, Thiadiazole), 139.10, 138.11, 137.80, 133.73, 130.15, 129.99, 129.51, 128.98, 127.28, 124.42, 124.20, 123.15, 121.61 (Arom.), 83.54 ppm (spiro). Anal. Calcd. for C_20_H_12_N_4_O_4_S (404.40): C, 59.40; H, 2.99; N, 13.85; S, 7.93% Found: C, 59.36; H, 2.95; N, 13.95; S, 7.91%.

##### Synthesis of 1,3'-diacetyl-2-oxo-*N*-phenyl-3'H-spiro[indoline-3,2'-[1,3,4]thiadiazole]-5'-carboxamide (8a)

2-Oxo-*N*-phenyl-3'H-spiro[indoline-3,2'-[1,3,4]thiadiazole]-5'-carboxamide **(6a)** (1.0 mmol), was refluxed in (10 mL) acetic acid for 3 h (monitored with TLC). After cooling, the solution was poured into ice water, the formed precipitate was filtered and crystallized from methanol.

Yellow crystals, yield 66%, mp. 225–227 ºC FT-IR (ATR) *ν*_max_: 3284 (NH), 1718, 1658, 1621, 1594 (C = O); ^1^H NMR: *δ* 10.58 (s, 1H, NH_amidic_, exchangeable by D_2_O), 8.13- 7.21 (m, 9H, ArH), 2.60 (s, 3H, CH_3_), 2.42 ppm (s, 3H, CH_3_); ^13^C NMR: *δ* 172.29, 170.71, 169.74, 156.82 (4C = O), 146.81 (C, Thiadiazole), 139.29, 137.70, 131.47, 129.30, 127.75, 126.49, 125.43, 124.97, 121.63, 116.17 (Arom.), 79.10 (spiro), 26.42, 22.14 ppm (2CH_3_). Anal. Calcd. for C_20_H_16_N_4_O_4_S (408.43): C, 58.81; H, 3.95; N, 13.72; S, 7.85% Found: C, 58.65; H, 3.97; N, 13.81; S, 7.92%.

##### General method for synthesis of compounds 9a and 10a

A mixture of 2-oxo-*N*-phenyl-3'H-spiro[indoline-3,2'-[1,3,4]thiadiazole]-5'-carboxamide **(6a)** (1.0 mmol), an appropriate methyl or ethyl iodide(1.0 mmol) and potassium carbonate (3.0 mmol) in acetone (10 mL) refluxed for 2 h (monitored with TLC). The formed precipitate was then filtered and crystallized from ethanol.

##### 1-Methyl-2-oxo-*N*-phenyl-3'H-spiro[indoline-3,2'-[1, 3, 4]-thiadiazole]-5'-carboxamide (9a)

Orange crystals, yield 92%, mp.233–235 ºC FT-IR (ATR) *ν*_max_: 3374, 3267 (2NH), 1726(C = O_isatin_), 1690(C = O_amidic_); ^1^H NMR: *δ* 10.37(s, 1H, NH_amidic_, exchangeable by D_2_O), 9.51 (s, H, NH_thiadiazole_, exchangeable by D_2_O), 7.74- 7.06 (m, 9H, ArH), 3.14 ppm (s, 3H, CH_3_); ^13^C NMR: *δ* 174.11 (C = O_isatin_), 158.04 (C = O_amidic_), 143.12 (C, Thiadiazole), 138.54, 138.35, 131.55, 129.11, 128.78, 125.93, 124.47, 123.93, 120.88, 109.67, (Arom.), 80.23 (spiro), 26.92 ppm (CH_3_). Anal. Calcd. for C_17_H_14_N_4_O_2_S (338.38): C, 60.34; H, 4.17; N, 16.56; S, 9.48% Found: C, 60.15; H, 4.32; N, 16.52; S, 9.55%.

##### 1-Ethyl-2-oxo-*N*-phenyl-3'H-spiro[indoline-3,2'-[1,3,4]thia-diazole]-5'-carboxamide (10a)

Orange crystals, yield 85%, mp. 220–222 ºC FT-IR (ATR) *ν*_max_: 3413, 3325 (2NH), 1713 (C = O_isatin_), 1667 (C = O_amidic_); ^1^H NMR: *δ* 10.29 (s, 1H, NH_amidic_, exchangeable by D_2_O), 9.51 (s, H, NH_thiadiazole_, exchangeable by D_2_O), 7.76- 7.09 (m, 9H, ArH), 3.74–3.69 (q, 2H, CH_2_), 1.21–1.18 ppm (t, 3H, CH_3_); ^13^C NMR: *δ* 173.50 (C = O_isatin_), 158.01 (C = O_thiadiazole_), 142.13 (C, Thiadiazole), 138.64, 138.24, 131.55, 129.08, 126.20, 124.38, 123.75, 120.85, 109.74 (Arom.), 80.21 (spiro), 35.09 (CH_2_), 12.78 ppm (CH_3_). C_18_H_16_N_4_O_2_S (352.41): C, 61.35; H, 4.58; N, 15.90; S, 9.10% Found: C, 61.41; H, 4.52; N, 15.93; S, 8.94%.

##### Synthesis of 6-bromo-2-oxo-*N*-phenyl-3'*H*-spiro[indoline-3,2'-[1,3,4]thiadiazole]-5'-carboxamide (11a)

A mixture of 2-oxo-*N*-phenyl-3'H-spiro[indoline-3,2'-[1,3,4]thiadiazole]-5'-carboxamide (**6a**) (1.0 mmol)and bromine (1.1 mmol) was heated in acetic acid at 60 ºC for 2 h. After cooling, the solution was poured into ice water, the formed precipitate was filtered and crystallized from ethanol.

Orange crystals, yield 90%, mp. 255–257 ºC FT-IR (ATR) *ν*_max_: 3378, 3342, 3239 (3NH), 1701 (C = O_cyclic_), 1680 (C = O_amidic_); ^1^H NMR: *δ* 10.68, 10.28 and 9.52 (s, 3H, 3NH, exchangeable by D_2_O), 7.74- 6.89 ppm (m, 8H, ArH); ^13^C NMR: *δ* 175.52 (C = O_cyclic_), 157.95 (C = O_amidic_), 140.45 (C, Thiadiazole), 138.60, 138.41, 131.63, 131.26, 129.09, 127.18, 126.20, 124.42, 120.87, 112.34 (Arom.), 80.42 ppm (spiro). Anal. Calcd. for C_16_H_11_BrN_4_O_2_S (403.25): C, 47.66; H, 2.75; Br, 19.81; N, 13.89; S, 7.95% Found: C, 47.61; H, 2.77; Br, 19.79; N, 13.92; S, 7.96%.

##### Synthesis of 3',3'''-(1-oxoethane-1,2-diyl)bis(2-oxo-*N*-phenyl-3'H-spiro[indoline-3,2'-[1,3,4]thiadiazole]-5'-carboxamide) (12a)

2-Oxo-*N*-phenyl-3'H-spiro[indoline-3,2'-[1,3,4]thiadiazole]-5'-carboxamide (**6a**) (1.0 mmol) and chloroacetyl chloride (1.0 or 0.5 mmol) was stirred in (10 mL) DMf for 5 h at room temperature (monitored with TLC). Then, the solution was poured into ice water, the formed precipitate was then filtered and crystallized from ethanol.

Orange crystals, yield 77%, mp. 283–285 ºC FT-IR (ATR) *ν*_max_: 3499, 3345, 3270, 3111 (4NH), 1771, 1716, 1655, 1688 (C = O); ^1^H NMR: *δ* 11.02, 10.59, 10.35 and 9.56 (s, 4H, 4NH, exchangeable by D_2_O), 7.96- 6.86 (m, 18H, ArH), 2.47 ppm (s, 2H, CH_2_); ^13^C NMR: *δ* 175.75, 164.29, 158.05 (C = O), 141.66 (C, Thiadiazole), 138.77, 137.86, 134.99, 131.44, 129.38, 128.66, 126.16, 123.93, 123.30, 120.82, 110.40 (Arom.), 80.18 (spiro), 56.40 ppm (CH_2_). Dept -135 NMR; 131.41, 129.08, 126.31, 124.38, 123.29, 120.85 (Arom.), 56.25 ppm (CH_2_).Anal. Calcd. For C_34_H_24_N_8_O_5_S_2_ (688.13): C, 59.29; H, 3.51; N, 16.27; S, 9.31% Found: C, 59.34; H, 3.49; N, 16.25; S, 9.30%.

### Assessment of in-vitro anti-arthritic activity

#### Inhibition of protein denaturation [25]

Two milliliters of synthetic chemicals at 50, 100, 200, and 300 µg/ml, two milliliters of puffer phosphate solution (pH 6.4), and two milliliters of egg albumin were included in the fivemilliliter reaction mixture. The normal solution contained indomethacin instead of a synthetic drug. The synthetic chemical solution was replaced in the control solution with distilled water. These solutions were heated to 70 °C for five minutes after an incubation period of fifteen minutes at 37 °C. The absorbance of the solutions at 660 nm was measured after they had warmed up to room temperature. The experiment was conducted in triplicate, and the percentage inhibition of denaturation of the protein was calculated using the following formula:

$$ {{\% }}\,{\mathrm{Inhibition}}\,\, = \,\frac{{{\mathrm{(Absorbance}}\,\,{\mathrm{Control}}{-}{\mathrm{Absorbance}}\,\,{\mathrm{Sample)}}\,\,{\mathrm{X}}\,100}}{{{\text{Absorbance control}}}} $$

IC_50_ value of synthesized compounds and standard were also calculated.

#### Effect on membrane stabilization

In this model, the anti-inflammatory activity was evaluated using the percentage of membrane stabilizing activity. Indomethacin served as the standard medication. The evaluation test was conducted using the methodology that Aidoo, Konja, et al. reported [26]. Three milliliters of fresh whole blood were drawn from healthy Wistar rats, and the blood was centrifuged for ten minutes at 3000 rpm. With the appropriate approval of the Institutional Animal Care and Use Committee (IACUC) of the Faculty of Science at Sohag University [Protocol Number: SU-FS-20–25], in accordance with international guidelines for the care and use of laboratory animals. The red blood pellets were dissolved in an equivalent volume of the supernatant in normal saline solution (0.9% NaCl). After the acquired dissolved red blood pellets were measured in volume, a 40% v/v suspension was prepared using an isotonic buffer solution (10 mM sodium phosphate buffer, pH 7.4). The buffer solution included 0.2 g of NaH_2_PO_4_, 1.15 g of Na_2_HPO_4_, and 9 g of NaCl in one liter of distilled water. In this manner, regenerated red blood cells (resuspended supernatant) were administered. To make a hypotonic solution, samples of produced chemicals were dissolved in distilled water. Duplicate pairs (per dose) of the hypotonic solution (5 ml) containing graded doses of the produced compounds (50, 100, 200, and 300 µg/ml) were placed inside the centrifuge tubes. Additionally, isotonic solution (5 ml) containing graded dosages of the produced compounds (50—300 µg/ml) was added to duplicate pairs (per dose) of centrifuge tubes. The drug of reference was indomethacin. The control tubes contained 5 ml of indomethacin 6 and 5 ml of the vehicle, which was distilled water. A gentle mixing of 0.1 ml of erythrocyte suspension was added to each tube. After being incubated for one hour at room temperature (37 °C), the mixtures were centrifuged for three minutes at a weight of 1300 g. The absorbance (OD) of the supernatant at 540 nm was measured for hemoglobin concentration using a Spectronic (Milton Roy) spectrophotometer. The proportion of hemolysis was determined by assuming that all hemolysis produced in the presence of distilled water is 100%. To calculate the amount of hemolysis [29] that the pyrimidine derivatives inhibited, the following formula was utilized:

$$ \begin{gathered} \% \,\,{\mathrm{Inhibition}}\,\,{\mathrm{of}}{\mkern 1mu} \,{\mathrm{hemolysis}}\,\, = {\mkern 1mu} \hfill \\ \left( {1 - \frac{{OD2 - OD1}}{{OD3 - OD1}}} \right)\, \times {\text{ 100}} \hfill \\ \end{gathered} $$

where OD1 is the test sample's absorbance in an isotonic solution.

- OD2 is the test sample's absorbance in a hypotonic solution.
- OD3 is the control sample's absorbance in a hypotonic solution.

Calculations were made for the IC50 value of the synthesized compounds and the standard for hemolysis inhibition.

#### Statistical analysis

Tukey's test was used after two-way analysis of variance (ANOVA) for statistical analysis of the results. Findings deemed noteworthy (p < 0.05) in relation to indomethacin.

### ADME profile

The SwissADME web tool [27] (https://www.sib.swiss/) was utilized to predict the ADME (Absorption, Distribution, Metabolism, and Excretion) profiles of studied compounds.

### Molecular docking

The molecular docking was done using Autodock vina [28]. The ligands, namely the most active potential six tested compounds (9a, 10a, 6a, 6d, 6b, and 6c) derived from practical data, underwent comprehensive molecular docking investigations utilizing (Insilco studies). The receptor in this study was the X-ray crystallographic structure of COX-2 with pdb ID 5IKT [29], obtained from the Protein Data Bank (https://www.rcsb.org/structure/5IKT). Prior to docking, polar hydrogen atoms were introduced to the target structures, and any extraneous components such as water molecules, native ligands, and undesired chains were eliminated [30]. Subsequently, the six tested compounds (9a, 10a, 6a, 6d, 6b, and 6c) were strategically docked within the active site of 5IKT [29].

The validation of the docking process was ensured through a meticulous strategy involving re-docking and superimposition [31–33]. In this procedure, the native ligand of the 5IKT enzyme was systematically removed and subsequently re-docked into the active site, detailed supplementary information is available in S.I. Docking Validation.

### Density functional theory (DFT) analysis

Conducting analyses through density functional theory (DFT) plays a pivotal role in computing molecular orbital properties. To delve into the structural aspects, the top three compounds (9a, 10a, and 6a) identified through the screening procedure underwent an extensive DFT analysis and B3LYP [34] with a 6-311G (d,p) [35] basis set using ORCA 5.1.0 [36]. A comparative examination was executed to scrutinize the energies of the highest occupied molecular orbital (HOMO) in relation to the lowest unoccupied molecular orbital (LUMO) [37, 38]. Various chemical reactivity characteristics, including energy gap (ΔE), chemical hardness (η), and softness (σ), were evaluated using HOMO–LUMO energies [39].

## Result and discussion

### Chemistry

Several thioxoacetamide derivatives were synthesized in excellent yields by reacting chloroacetanilides with sulfur and morpholine, followed by treatment with hydrazine, according to established literature procedures [40, 41].

**Scheme 1:**
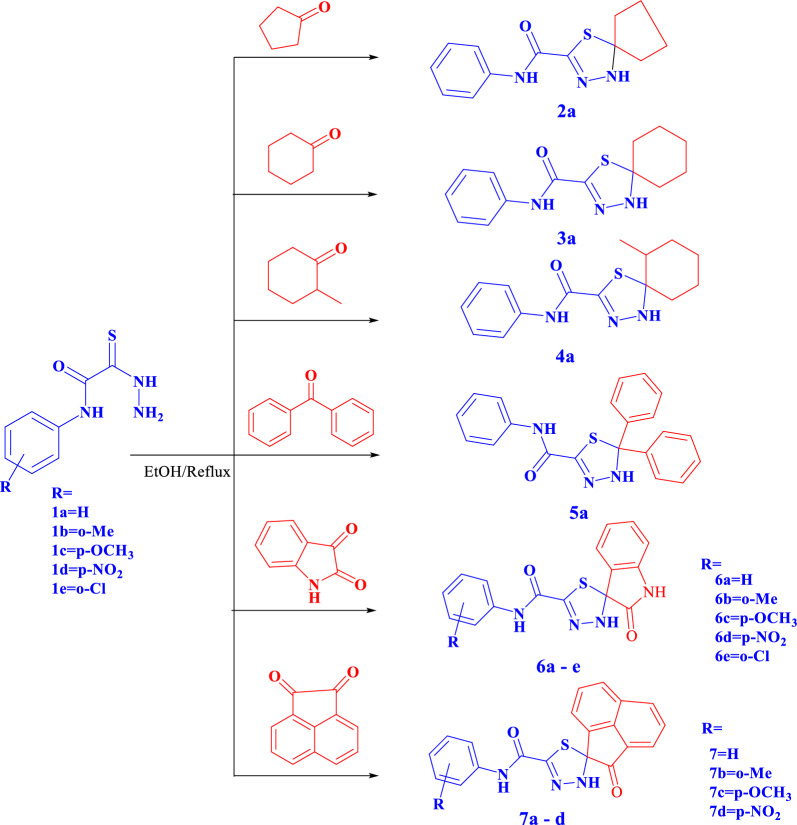
Synthesis of spiro-1,3,4-thiadiazole derivatives

In continuous of our work in the synthesis of some new spiroheterocyles [26, 42–46] we investigate the reaction of 2-hydrazinyl-*N*-phenyl-2-thioxoacetamides **1a-e** with some reagents namely; cyclopentanone, cyclohexanone, 2-methylcyclohexanone, benzophenone, isatin and acenaphthylene-1,2-dione (see Scheme 1) to give novel 1,3,4-spirothiadiazole compounds **2a,3a,4a,6a-e**and **7a-d**, respectively. In addition to the reaction with** 1** benzophenone afforded 1,3,4-thiadiazole compounds **5a**.

The chemical structures of recently synthesized compounds were proved by their spectral (IR, ^1^H, ^13^C NMR), as well as elemental analysis, (see **Experimental part)**.

IR spectra for compounds **2a, 3a** and **4a** showed the disappearance of NHNH_2_ group absorption bands and appearance a band of NH groups between 3441 and 3241 cm^−1^ and carbonyl groups between 1680 and 1667 cm^−1^. Their^1^H-NMR spectra showed signal at 10.32, 10.05, 10.36 ppm for NH_amidic_ and at 8.93, 8.66, 8.77 ppm for NH_cyclic_ groups (disappeared by D_2_O), respectively, between 7.80–6.98 ppm for aromatic protons and new signals at 2.22–1.00 ppm for the cyclopentanone and cyclohexanone ring, furthermore a new signal at 0.98 ppm for methyl group for compounds **4a.** Thier^13^CNMR spectraexpected structure by appearance new signals at 80.46, 87.68 and 79.14 ppm characterized to spiro carbon atoms respectively, in addition to appearance of new signals in the aliphatic region characterized to cyclopentanone and cyclohexanone ring.Dept-135 spectra also showed new signals at 26.17, 24.66 ppm and 30.63, 24.98, 24.27 ppm in the negative phase for cyclopentanone and cyclohexanone ring, respectively.

For compound **5a,** IR spectra illustrated the appearance of bands at 3454, 3343 cm^−1^ characterized to NH groups. Their^1^H-NMR spectra showed new signals at11.21 ppm and 10.67 ppm for two NH groups (disappeared by D_2_O), between 8.16–6.66 ppm for aromatic rings. Its^13^CNMR spectrumsignals confirmed the expected structure by increasing the signals of the aromatic carbon atoms, furthermore appearance of new signals 75.58 ppm characterized for spiro carbon atom.

IR spectra of compounds **6a-e** and **7a-d** showed the appearance of the absorption bands of NH_thiadiazole_ groups, furthermore appearance of characteristic bands owing to carbonyl groups between 1724 and 1667 cm^−1^, moreover the nitro group appeared at1330-1535 cm^−1^ for compound **6d.** In addition to appearance of singlet signals for NH_amidic_ and NH_thiadiazole_, the ^1^H-NMR spectrum for compound **6a-e** showed appearance of new exchangeable NH_isatin_ at 9.51, 9.50, 9.55, 9.79 and 9.64 ppm respectively. In addition to appearance of signals for spiro carbon atom, ^13^C NMR spectra showed presence of new signals for C = O_isatin_ at 175.75, 175.80, 175.83 and 175.38 ppm, respectively, whereas ^1^H-NMR spectra for compounds **7a-d** showed singlet signals at 10.37–10.60 ppm for NH_amidic_ and at 9.21–9.73 ppm for NH_cyclic_ group (disappeared by D_2_O), also there is increasing in the number of protons in the aromatic region. Their ^13^CNMR spectra signals confirmed the expected structure by appearance of new signals at 198.73, 195.62, 198.86, 199.55 ppm, respectively, for cyclic carbonyl group. (Scheme 1**).**

**Scheme 2:**
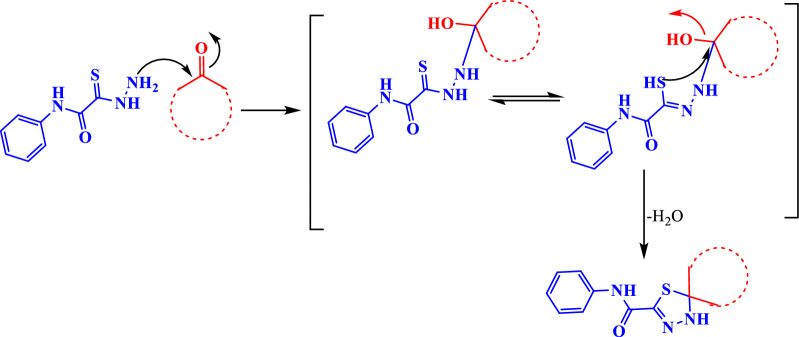
Reaction mechanism for spiro-1,3,4-thiadiazole derivatives

The reaction mechanism was assumed via a nucleophilic attack of amino group of thiocarbohydrazide **1** at the carbonyl group of ketone followed via a nucleophilic attack of hydrazine group at the carbonyl carbon of ketone to afford thiohydrazone followed by a nucleophilic attack of thiole group at the same carbonyl group of ketone with elimination of water and cyclization (Scheme 2).

Nevertheless, the reaction of compound **6a** with acetic anhydride gave 1,3'-diacetyl-2-oxo-*N*-phenyl-3'*H*-spiro[indoline-3,2'-[1,3,4]thiadiazole]-5'-carboxamide (**8a**).IR spectrum for compound **8a,** showed characteristic absorption bands at 3284 cm^−1^ for NH group and 1718, 1658 and 1621 cm^−1^carbonyl groups; Its ^1^H NMR spectrum illustrated singlet signal at 10.58 ppm for NH_amidic_, exchangeable by D_2_O, multiplet signals between 8.13–7.21 ppm for aromatic protons and singlet signals at 2.60 and 2.42 ppm for two methyl groups. Its ^13^C NMR the appearance of new signals at 172.29, 170.71, 169.74, 156.82 ppm for carbonyl groups, 146.81 ppm C = N_thiadiazole_, 139.29, 137.70, 131.47, 129.30, 127.75, 126.49, 125.43, 124.97, 121.63, 116.17 ppm for aromatic carbons, 79.10 ppm for spiro carbon atom, 26.42 and 22.14 ppm for 2CH_3_ groups.

On treatment of compound **6a** with alkyl halides namely: methyl iodide, ethyl iodide, yielded spiro[indoline-3,2'-[1, 3, 4]-thiadiazole]-5'-carboxamide derivatives **9a** and **10a** respectively.

**Scheme 3.**
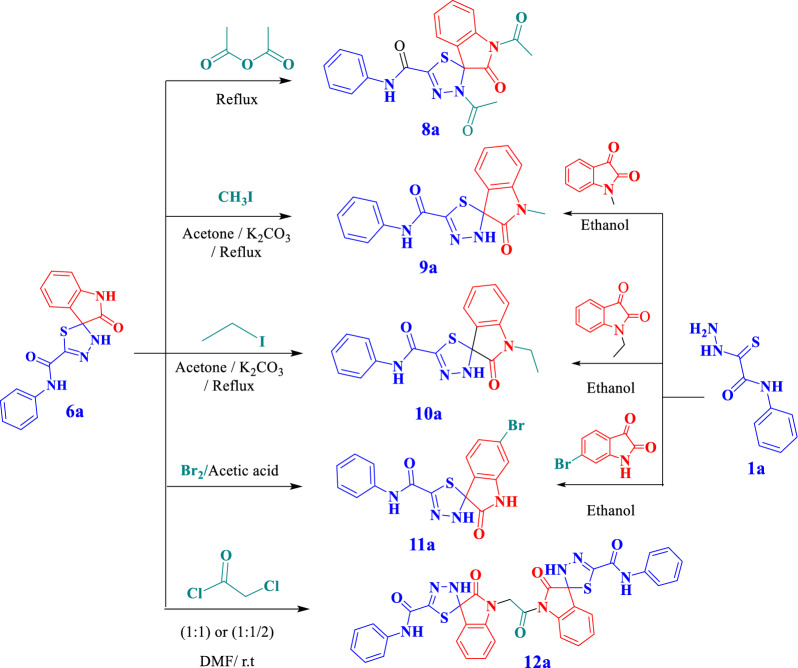
Synthesis of 2-oxo-*N*-phenyl-3'*H*-spiro[indoline-3,2'-[1,3,4]thiadiazole]-5'-carboxamide Derivatives

Bromination of compound **6a** with bromine/acetic acid afforded 6-bromo-2-oxo-N-phenyl-3'H-spiro[indoline-3,2'-[1,3,4]thiadiazole]-5'-carboxamide **(11a)**. Also, 3',3'''-(1-Oxoethane-1,2-diyl)bis(2-oxo-*N*-phenyl-3'*H*-spiro[indoline-3,2'[1,3,4]thiadiazole]-5'-carboxamide) **(12a)** was obtained via reaction of compound **6a** with chloroacetyl chloride (1:1 or 1:0.5). Another method was performed via reaction of compound **1a** with *N*-methylisatin, *N*-ethyl isatin, or 5-bromoisatin to confirm the product structures **9a, 10a** and **11a,** respectively **(**Scheme 3**).**

For compounds **9a** and **10a**, Thier^1^H-NMR spectra showed new signals at 3.14 ppm for the methyl group for compound **9a,** 3.74–3.69 ppm for methelene group and at 1.21–1.18 ppm for methyl group for compound **10a**. Their^13^CNMR spectrasignalsconfirmed the expected structure by appearance of new signals at 174.11, 173.50 ppm for carbonyl groups, 80.23, 80.21 ppm for spiro quaternary carbon atom, respectively, and 26.92 ppm for methyl group for compound **9a**, at 35.09 ppm and 12.78 ppm characterized for ethyl group of compounds **10a**.

Moreover, ^1^H-NMR spectrum of compound **11a**illustrated eight multiple signals between 7.74- 6.89 ppm for aromatic protons. Its ^13^C NMR spectrum showed signals at 175.52, 157.95 ppm for two carbonyl groups and at 80.42 ppm for spiro carbon atom.

For compound **12a**, its ^1^H-NMR spectrum showed signals at 11.02, 10.59, 10.35 and 9.56 ppm for 4NH groups (disappeared by D_2_O), multiplet signals between 7.96–6.86 ppm for aromatic protons and a new signal at 2.47 ppm for the methylene group. It's ^13^CNMR spectrum signals confirmed the expected structure by appearance of new signals at 175.75, 164.29, and 158.05 ppm for carbonyl groups, at 80.18 ppm for spiro quaternary carbon atom, and at 56.40 ppm for methylene group. Its Dept-135 spectrum illustrated the methylene group in the opposite direction at 56.25 ppm**.**

Finally, These syntheses are not random—they are hypothesis-driven structural modifications aimed at:

1. Mapping the SAR of the spirothiadiazole scaffold
2. Identifying key pharmacophores for COX-2 inhibition and membrane stabilization
3. Providing a foundation for lead optimization in future studies

This approach is both scientifically and strategically aligning with modern medicinal chemistry practices. To further explore the structure–activity relationship was selectively functionalized:

(i) N-Alkylation (methyl, ethyl) afforded **9a** and **10a** to probe the role of the isatin N–H group which Marked improvement in activity (especially 9a, the most potent compound);
(ii) Bromination yielded **11a** to assess the impact of aromatic halogenation where Reduced activity, guiding future design away from bulky halogens at this position;
(III) Dimerization via a chloroacetyl linker gave **12a** to evaluate multivalent binding potential.

Additionally, compounds **9a–11a** were also synthesized independently from precursor **1a** by using two synthetic routes to.

- Confirms structures by independent synthesis
- Validates the reaction mechanism
- Ensures that observed activities are due to the intended structures

This dual-route approach is a mark of rigorous synthetic chemistry, ensuring reproducibility and structural certainty.

### Biological activity

#### Evaluation of anti-arthritic activity using *in-vitro* model

##### Effect on protein denaturation and membrane stabilization

**Fig. 2:**
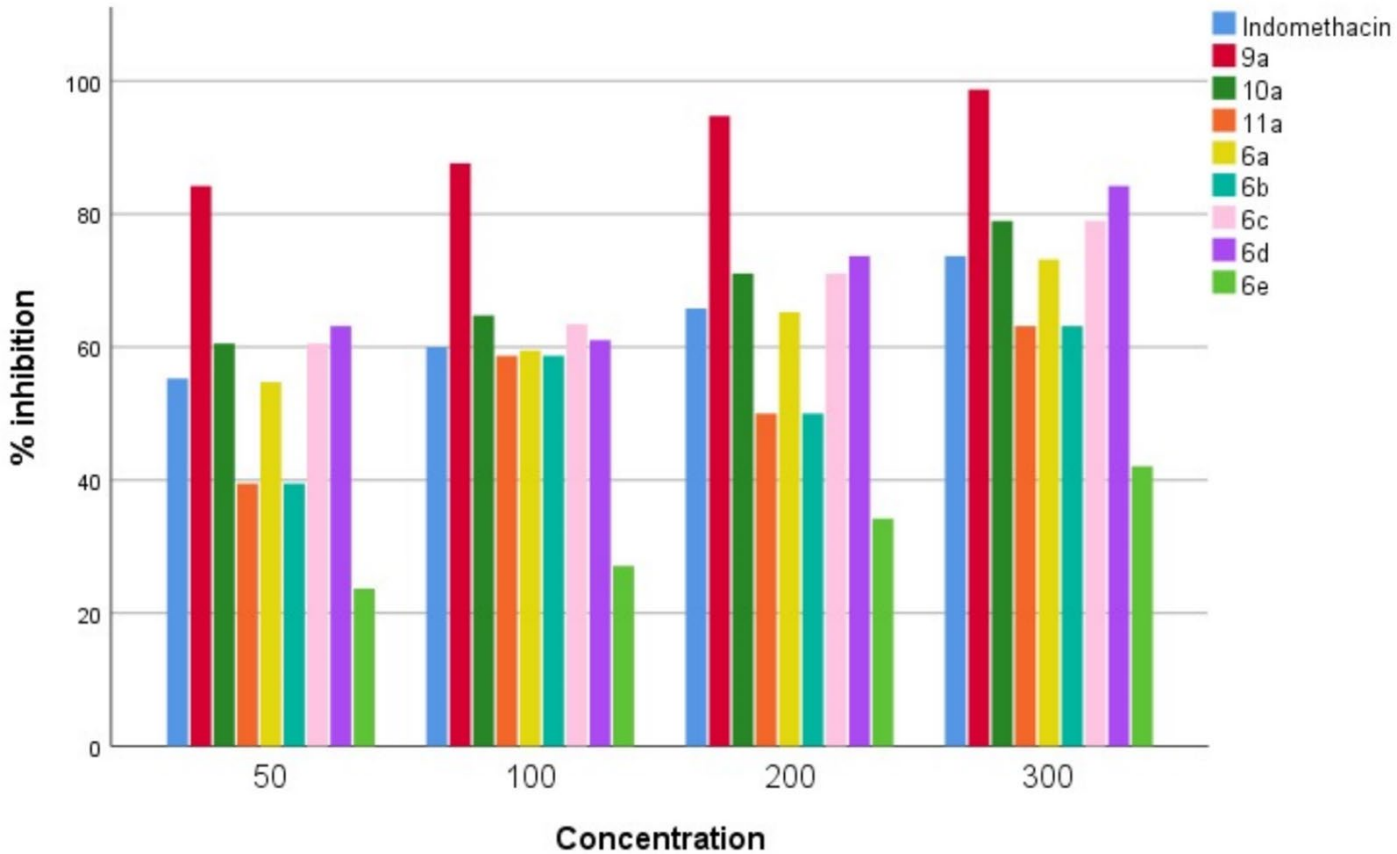
In vitro anti-arthritic activity by protein denaturation

**Fig. 3:**
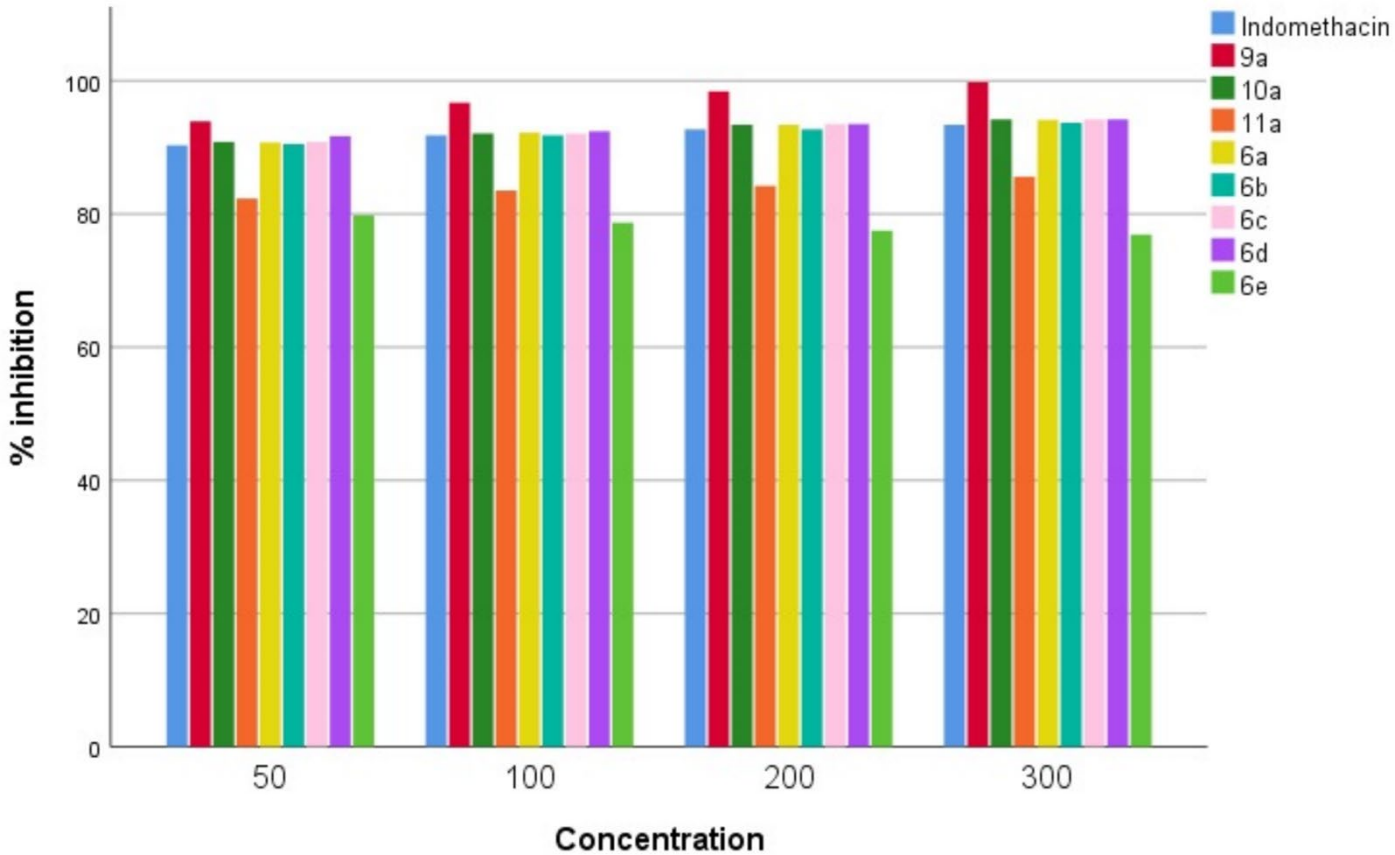
In vitro anti-arthritic activity by membrane stabilization

The percentage inhibition of egg albumin denaturation and stabilization the RBC membrane was maximum with synthesized compounds **9a, 10a, 6a, 6b, 6c**, and **6d** at 50-300 µg/ml. All synthesized compounds impeded protein denaturation dose dependently with remarkable effect and maximum inhibition of RBC membrane lysis as mentioned in (Fig. 2, 3). The value of IC50 of percentage inhibition of egg albumin denaturation and stabilization of synthesized compounds was demonstrated in Table 1**.**

**Table 1:** In vitro anti-arthritic activity of synthesized compounds by protein denaturation and membrane stabilization methods

Compound Code	Conc. (µg/ml)	% inhibition of protein denaturation	IC_50_ (µg/ml) for protein denaturation	% inhibition of RBC hemolysis	IC_50_ (µg/ml) for RBC hemolysis
Indomethacin	50 100 200 300	55.26 60.0 65.78 73.68	83.3	90.3 91.8 92.7 93.4	27.6
**9a**	50 100 200 300	84.21 87.63 94.73 98.68	29.6	93.9 96.7 98.4 99.8	26.6
**10a**	50 100 200 300	60.52 64.73 71.05 78.94	41.3	90.8 92.1 93.4 94.2	27.5
**11a**	50 100 200 300	39.47 58.68 50.0 63.15	63.3	82.3 83.5 84.2 85.6	30.3
**6a**	50 100 200 300	60.52 63.94 71.05 78.94	41.3	90.7 92.2 93.4 94.1	27.5
**6b**	50 100 200 300	57.89 64.21 71.05 78.94	43.1	90.5 91.8 92.7 93.7	27.6
**6c**	50 100 200 300	60.52 63.42 71.05 78.94	41.3	90.8 92.1 93.5 94.2	27.5
**6d**	50 100 200 300	63.15 61.05 73.68 84.21	39.5	91.7 92.4 93.5 94.2	27.2
**6e**	50 100 200 300	23.68 27.10 34.21 42.10	105.5	79.8 78.7 77.5 76.9	31.3

IC_50_ value is the compound concentration required to produce 50% inhibition

All the values are significant when compared to indomethacin (p < 0.05)

In the current work, strategies to prevent protein denaturation and stabilise membranes were used to investigate the in-vitro anti-arthritic activity of synthesised compounds.

Proteins can become denaturized under pressure from heat and chemical exposure, which leads to the production of autoantigens that damage cartilage and the synovial membrane in joints [47]**.** The denaturation of egg albumin at 50, 100, 200, and 300 g/ml was prevented by all synthetic substances. The highest activity explored by **9a**, **10a, 6a, 6b, 6c,** and **6d** than other tested compounds and standard drug indomethacin.

In cases of arthritis, lysosomal membrane lysis results in the leakage of enzymes such proteases and phospholipase A2. Lysosome-like membranes can be found on RBCs. Consequently, substances that protect RBC membrane from damage may also be able to stabilise endogenous lysosomal membrane [48]. The results of the study showed that synthetic compounds were more resistant to RBC lysis than the indomethacin. Compounds **9a, 10a, 6a, 6b, 6c, and 6d** surpassed indomethacin in terms of membrane protection and strong anti-inflammatory actions, making them more effective in preventing lysis.

### ADME profile

The SwissADME analysis indicates that most of the studied compounds exhibit favorable pharmacokinetic profiles, Table 2. The calculated topological polar surface area (TPSA) values range from 99.10 to 153.71 Å^2^, suggesting that compounds 6a, 6b, 6c, 9a, and 10a fall within the acceptable range for oral bioavailability, whereas compound 6d, with a TPSA of 153.71 Å^2^, shows reduced gastrointestinal absorption, in agreement with its predicted low GI absorption.

**Table 2:** Pharmacokinetic properties and drug-likeness parameters of the studied compounds calculated using the SwissADME web tool

Compound	TPSA (Å^2^)	XLOGP3	WLOGP	GI absorption	BBB permeant	P-gp substrate	CYP2D6 inhibitor	Log Kp (cm s^−1^)	Lipinski violations
**6a**	107.89	2.69	0.45	High	No	Yes	Yes	−6.37	0
**6b**	107.89	3.05	0.76	High	No	Yes	Yes	−6.20	0
**6c**	117.12	2.66	0.46	High	No	Yes	Yes	−6.57	0
**6d**	153.71	2.52	0.88	Low	No	Yes	No	−6.76	0
**9a**	99.1	2.87	0.66	High	No	No	Yes	−6.33	0
**10a**	99.1	3.24	1.05	High	No	No	Yes	−6.15	0

All compounds display moderate lipophilicity, with XLOGP3 values between 2.52 and 3.24, which is consistent with balanced membrane permeability and solubility. None of the compounds are predicted to cross the blood–brain barrier (BBB), indicating a reduced risk of central nervous system side effects [49].

Compounds 6a-6d are predicted to be P-glycoprotein substrates, which may affect their intestinal efflux and bioavailability, while compounds 9a and a10a are non-substrates, suggesting potentially improved absorption profiles. With respect to metabolic interactions, all compounds except 6d are predicted to inhibit CYP2D6, which should be considered in the context of possible drug-drug interactions.

The predicted skin permeation coefficients (log Kp) fall within a narrow range (-6.76 to -6.15 cm s^−1^), indicating low skin permeability for all compounds. Importantly, no Lipinski rule violations were observed, supporting the overall drug-likeness of the investigated molecules.

### Molecular docking

The molecular docking procedure entails the anticipation of the most favorable orientation and binding affinity between a ligand, represented by the seven most potent compounds (**9a, 10a, 6a, 6d, 6b**, and **6c**) derived from experimental data, and a receptor, specifically the 5IKT molecule. This process culminates in the establishment of a stable complex, emphasizing the critical role of molecular interactions in ligand-receptor binding [50].

The significance of 5IKT is underscored by its capacity to furnish molecular insights into the mechanisms by which the tested compounds act as inhibitors of COX-2 [42, 44]. This holds promising implications for the development of novel pharmaceuticals aimed at targeting COX-2 for the treatment of diverse diseases [42, 44, 45]. Moreover, the exploration of 5IKT may contribute to an enhanced understanding of the role played by COX-2 in mediating the effects of endocannabinoids, signaling molecules that intricately regulate various biological functions including pain, mood, appetite, and memory [42, 44, 45]. In the broader context, molecular docking emerges as a potent tool for exploration, discovery, and design of pharmaceutical agents with specific targeting capabilities towards particular enzymes [43, 51, 52].

**Fig. 4.**
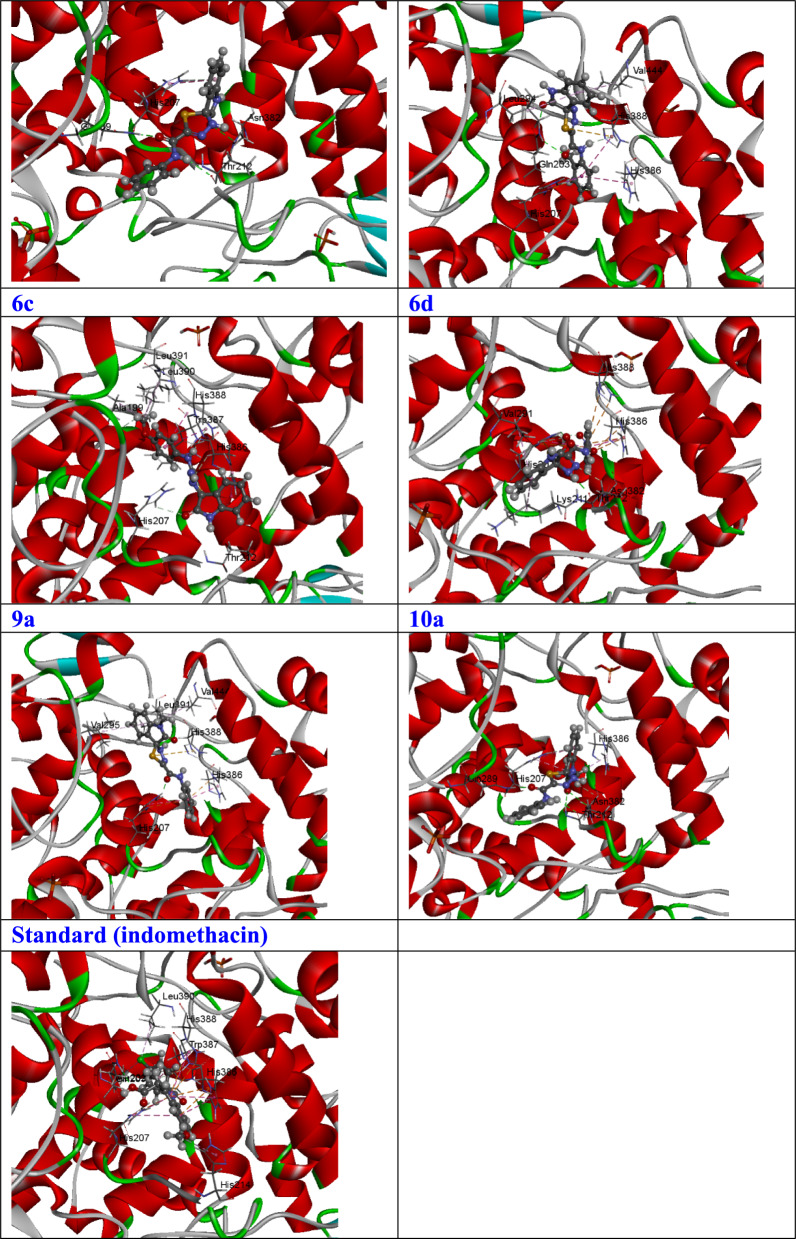
3D representations of the molecular interactions of the most active potential four tested compounds (**6a, 6b, 6c, 6d, 9a,**and **10a**) from practical data against human cyclooxygenase-2 (COX-2) (PDB ID: 5IKT)

In the preliminary stage, the docking protocol underwent validation through re-docking and superimposition processes. The re-docking approach employed identical procedures as in the initial setup. Examination revealed that the re-docked 5IKT native ligand perfectly overlapped with the native co-crystallized ligand, as illustrated in Fig. 4**.** The utilization of the re-docking and superimposition protocol provided robust evidence substantiating the validation of the docking procedure [31–33].

Table 3 provides the binding energys for the investigated compounds (specifically, the four most promising compounds: **9a, 10a, 6a, 6d, 6b**, and **6c**) against the human cyclooxygenase-2 (COX-2) enzyme, identified by the Protein Data Bank (PDB) ID 5IKT. The binding energys (S) exhibited a range between − 8.59 (for compound 9a) and − 7.29 (for compound 6c) kcal/mol. Notably, compounds 9a, 10a, and 6a displayed the highest binding affinities with binding energys of − 8.59, − 8.45, and − 8.05 kcal/mol, respectively, while compounds 6d (S = − 7.93 kcal/mol) and 6b (S = − 7.82 kcal/mol) followed closely. Compound 6c demonstrated the least activity with a binding energy of − 7.29 kcal/mol. Figure 4 visually represents the precise binding positions of these compounds within the active site of the 5IKT in both three-dimensional (3D) and two-dimensional (2D) formats. Additionally, Table 3 provides comprehensive docking data associated with each of these compounds.

**Table 3:** Binding energys of the most active potential six tested compounds (**6a, 6b, 6c, 6d, 9a,** and **10a**) from practical data against human cyclooxygenase-2 (COX-2) (PDB ID: 5IKT)

	Ligand	Receptor	Interaction	Distance	S (kcal/mol)	%
**6a**	N 7	THR 212	H-bond	3.38	−8.05	92.53
	O 9	GLN 289	H-bond	3.02		
	6-ring	HIS 207	pi-cation	3.91		
**6b**	N 7	HIS 388	H-bond	3.00	−7.82	89.89
	O 23	GLN 203	H-bond	2.92		
	6-ring	HIS 386	pi-H	4.45		
**6c**	N 7	HIS 388	H-bond	3.06	−7.29	83.79
	N 12	HIS 207	H-bond	3.01		
	6-ring	TRP 387	pi-H	4.28		
**6d**	N 12	THR 212	H-bond	2.87	−7.93	91.15
	O 9	HIS 207	H-bond	3.02		
**9a**	N 7	HIS 388	H-bond	3.07	−8.59	98.74
	O 9	HIS 207	H-bond	2.83		
	6-ring	HIS 386	pi-pi	3.80		
**10a**	N 7	THR 212	H-bond	3.41	−8.45	97.13
	C 20	HIS 388	H-bond	3.39		
	O 9	GLN 289	H-bond	2.98		
	6-ring	HIS 207	pi-cation	3.75		
**St**	CL 1	ALA199	H-bond	3.01	−8.70	
	O 4	GLN203	H-bond	2.84		

During the scrutiny of molecular interactions involving compound **9a**, it is evident that one hydrogen-donor interaction is established between N7 and HIS388, one hydrogen-acceptor interaction between O9 and HIS207, and one pi–pi interaction between the 6-ring and HIS386, occurring at distances of 3.07, 2.83, and 3.80 Å, respectively. The specifics of these interactions are concisely presented in Table 3**.**

Concerning compound **10a**, interactions include two hydrogen-donor interactions between N7 and THR212, one hydrogen-acceptor interaction between C20 and HIS388, one hydrogen-donor interaction between O9 and GLN289, and one pi-cation interaction between the 6-ring and HIS207, with respective distances of 3.41, 3.39, 2.98, and 3.75 Å, as comprehensively outlined in Table 2**.** In the case of compound **6a**, interactions are characterized by one hydrogen-donor interaction between N7 and THR212, one hydrogen-acceptor interaction between O9 and GLN289, and one pi-cation interaction between the 6-ring and HIS207, with distances measuring 3.38, 3.02, and 3.91 Å, respectively, as elucidated in Table 3**.**

Examining compound **6d** reveals the presence of one hydrogen-donor bond and one hydrogen-acceptor bond, with respective distances of 2.87 and 3.02 Å, established between N12 and THR212, and O9 and HIS207, as detailed in Table 2. Compound **6b**, on the other hand, exhibits one hydrogen-donor interaction, one hydrogen-acceptor interaction, and one pi-cation interaction between N7 and HIS388, O23 and GLN203, and the 6-ring and HIS386, respectively. These interactions occur at distances of 3.00, 2.92, and 4.45 Å, as presented comprehensively in Table 3.

Contrastingly, in the case of compound **6c**, a single hydrogen-donor interaction, a hydrogen-acceptor interaction, and a pi-cation interaction are observed. Specifically, these interactions occur between N7 and HIS388, N12 and HIS207, and the 6-ring and TRP387, respectively, with distances of 3.06, 3.01, and 4.28 Å. These detailed molecular interactions are presented comprehensively in Table 3.

The percentage efficiency (% = (S_test_/S_standard_) * 100) of the investigated compounds was assessed in comparison to the standard drug Indomethacin, as detailed in Table 2. Indomethacin exhibited one hydrogen-donor bond between Cl1 and ALA199, and one hydrogen-acceptor bond between O4 and GLN203, with distances of 3.01 and 2.84, respectively, accompanied by a high binding energy of -8.70 kcal/mol. Intriguingly, the title compounds displayed noteworthy percentage efficiencies (%), ranging from 98.74% (for **9a**), 97.13% (for **10a**), and 92.53% (for **6a**), to 83.79% (for **6c**), when compared to Indomethacin as the standard drug, as outlined in Table 2.

### Density functional theory (DFT) analysis

**Fig. 5:**
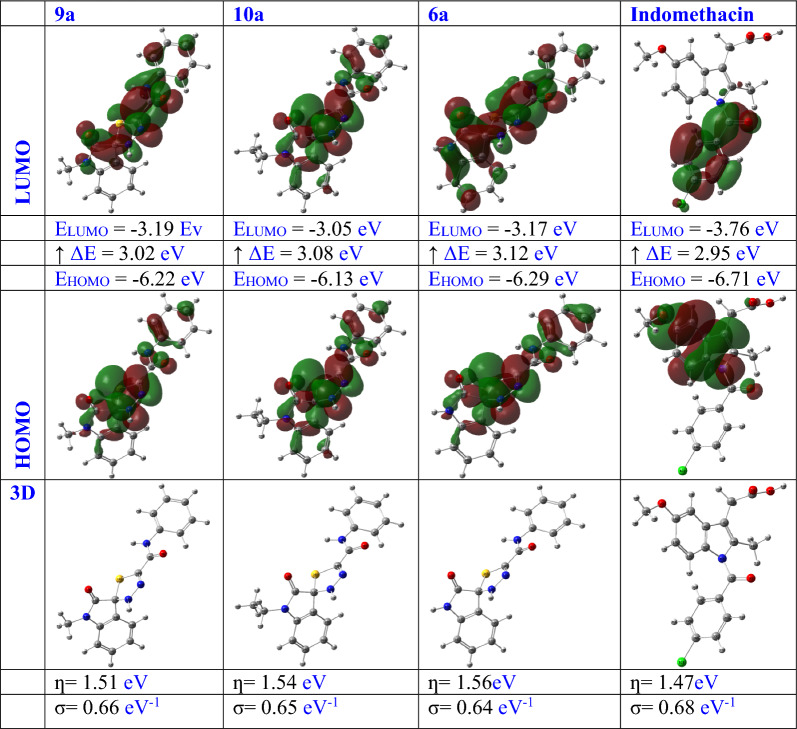
DFT exploration of compounds (**9a, 10a, 6a**) and reference drug Indomethacin

The DFT analysis employed the B3LYP [34] with a 6-311G (d,p) [35] basis set. Figure 5 illustrates the DFT evaluations of the three top-scoring compounds (**9a, 10a**, and **6a**) identified through the screening process, alongside the reference medication indomethacin. The HOMO–LUMO energy differences (ΔE) for compounds 9a (3.02 eV), 10a (3.08 eV), and 6a (3.17 eV) were observed to be similar to that of indomethacin (2.95 eV), as indicated by the DFT analysis results. This finding underscores the significance and potential of molecular charge transfer [53]. Furthermore, the energies of the HOMO and LUMO orbitals for the three top-ranked compounds (**9a, 10a**, and **6a**) identified in the screening process, as well as the reference medication indomethacin, were assessed to determine the hardness (ƞ, eV) and softness (σ, eV^−1^) values [46]. Notably, compounds 9a, 10a, and 6a exhibited chemical hardness and softness values comparable to those of indomethacin (0.68 eV^−1^, 1.47 eV, and 0.64 eV^−1^, respectively, versus 1.51 eV, 1.54 eV, and 1.56 eV, respectively). This observation could offer insights into why the investigated compounds displayed relatively high chemical reactivity compared to indomethacin.

### Structure–activity relationship (SAR) of the synthesized spirothiadiazole derivatives

**Fig. 6:**
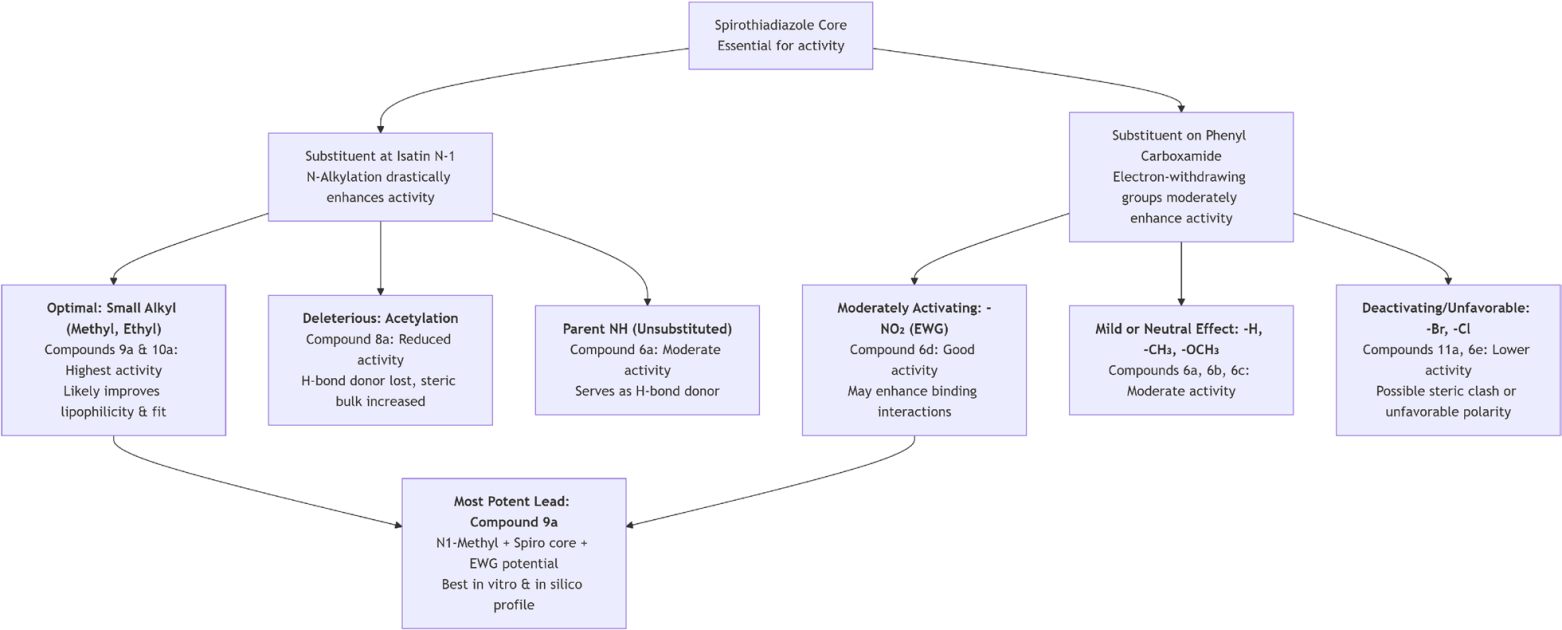
Structure–activity relationship (SAR) of the synthesized spirothiadiazole derivatives

A distinct Structure–Activity Relationship (SAR) is shown based on the biological evaluation of the produced spiro[1,3,4]thiadiazole derivatives (protein denaturation inhibition, membrane stability, and COX-2 docking). The core spiro structure, substituents on the isatin nitrogen (N-1), and substituents on the phenyl carboxamide ring all affect the activity. The main structural elements influencing anti-arthritic action are highlighted in the graphical SAR summary that follows in Fig. 6**.**

A spiro[1,3,4]thiadiazole-indolinone core replaced with a minor alkyl group (methyl/ethyl) at the isatin N-1 position such as compound **9a** and **10a** and an electron-withdrawing group (like NO₂) on the terminal phenyl ring yields the most powerful anti-arthritic profile in this series such as compound **6d**. Protein denaturation and membrane lysis are better inhibited by this combination because it maximizes lipophilicity, steric fit, and important intermolecular interactions (H-bonding, π-stacking) with the COX-2 enzyme.

## Conclusion

In summary, a series of novel spiro[1,3,4]thiadiazole derivatives were successfully synthesized and characterized. Additionally, thioxoacetamide reacts with a number of chemicals that have electrophilic centres, including cyclopentanone, cyclohexanone and isatin…etc. to for spirothiadiazoles. The synthesis of title compounds in this study has been accomplished by a preparation method that reduces reaction time, simplifies operation, and facilitates quick work-up. Every spectroscopic examination verified the suggested structures for these substances primary causes for treatment of rheumatoid arthritis. The findings suggest that the synthesized chemicals might regulate the synthesis of autoantigens by limiting protein denaturation. One of the ways that anti-inflammatory action is contributed is by preventing lysosomal membrane lysis. The study's synthesized materials showed a preventive effect against hypotonicity-induced RBC membrane lysis. The lysosomal membrane, which is identical to the RBC membrane, may therefore be stabilised by the synthetic chemicals, leading to the induction of an anti-inflammatory action. A clear road map for future lead optimization is provided by this SAR analysis, which focuses on investigating N-alkyl groups and phenyl ring replacements to create even more effective and specific anti-arthritic drugs. Future work will focus on *in-vivo* studies and structural optimization to enhance potency and selectivity.

## Supplementary Information

Supplementary file 1.

## Data Availability

“The datasets used and/or analysed during the current study are available from the corresponding author on reasonable request.”
