# Supplementary material for: Synthesis, characterization, molecular docking and in vitro anti-arthritic activity of some novel spiro [1,3,4] thiadiazole derivatives based on thioxoacetamides
Source: BMC Chem. 2026 Mar 8;20(1):52. doi: 10.1186/s13065-026-01745-w (PMC12969920; doi:10.1186/s13065-026-01745-w)
Supplement: Supplementary file 1 [file 13065_2026_1745_MOESM1_ESM.docx]

**Synthesis, Characterization, *Molecular Docking* and *In Vitro* Anti-Arthritic Activity of Some Novel Spiro[1,3,4]thiadiazole Derivatives Based on Thioxoacetamides**

Ahmed M. El-Saghier*^a^, Asmaa Abdul-Baset^a^, Omer M. El-Hady^a^, Aly Abdou^a^, Amany M. Hamed^a^ and Asmaa M. Kadry^a^

^a)^ *Chemistry Department, Faculty of Science, Sohag University, Sohag, Egypt.* [el.saghier@science.sohag.edu.eg](mailto:el.saghier@science.sohag.edu.eg)

Ahmed M. El-Saghier : [*el.saghier@science.sohag.edu.eg*](mailto:el.saghier@science.sohag.edu.eg) ; [*el_saghier@yahoo.com*](mailto:el_saghier@yahoo.com)

Asmaa Abdul-Baset : [asmaa2016044@science.sohag.edu.eg](mailto:asmaa2016044@science.sohag.edu.eg)

Omer M. El-Hady : [el7ady_omar@yahoo.com](mailto:el7ady_omar@yahoo.com)

Aly Abdou: [aly_abdou@science.sohag.edu.eg](mailto:aly_abdou@science.sohag.edu.eg)

Amany M. Hamed: [amanymohamed@science.sohag.edu.eg](mailto:amanymohamed@science.sohag.edu.eg)

Asmaa M. Kadry : [asmaa.kadry@science.sohag.edu.eg](mailto:asmaa.kadry@science.sohag.edu.eg)

Figure of supplemental 1: IR of compound 2a.


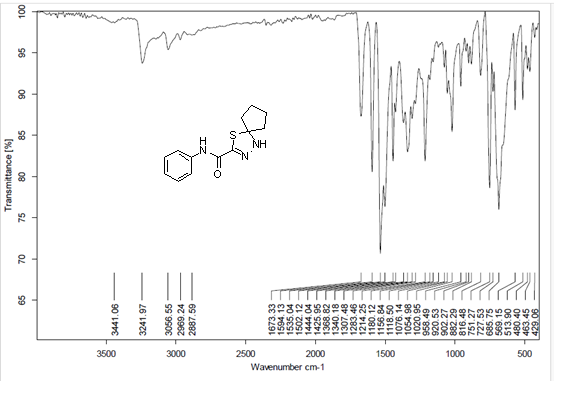


Figure of supplemental 2: ^1^HNMR of compound 2a.


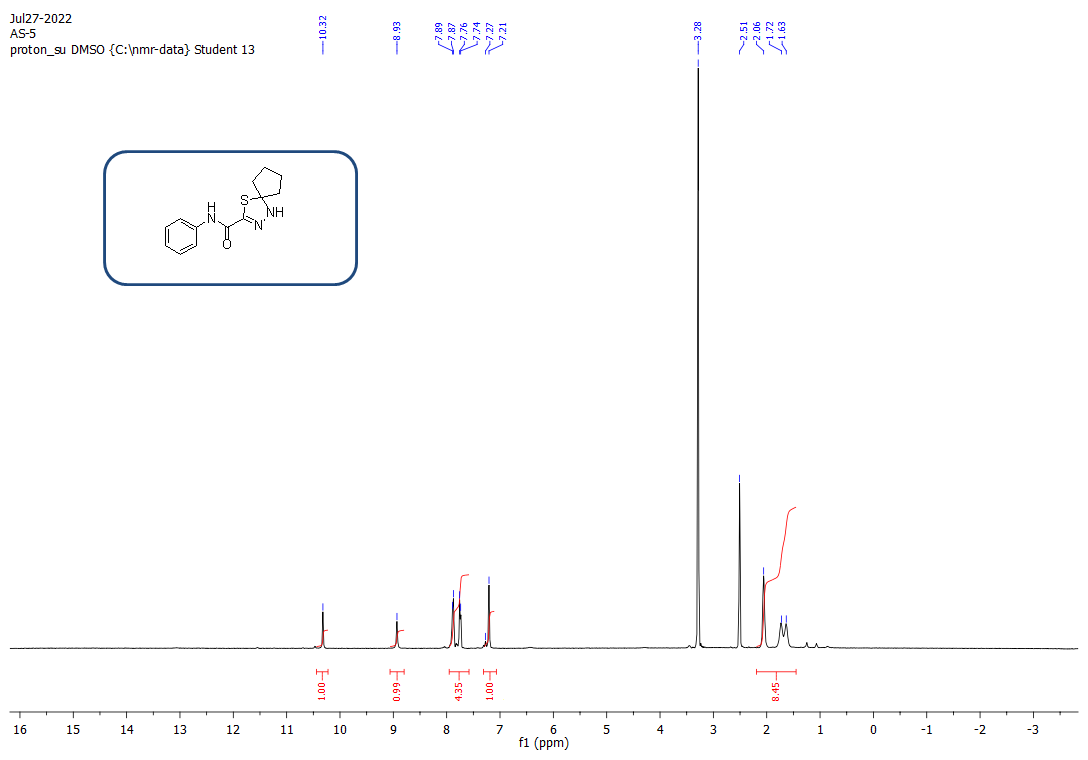


Figure of supplemental 3: ^13^C NMR of compound 2a.

**
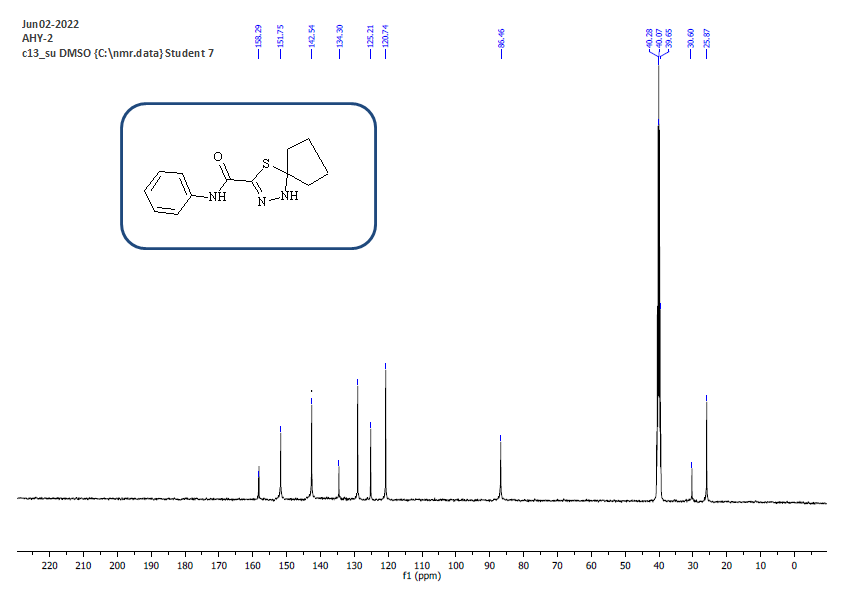
**

Figure of supplemental 4: Dept 135 of compound 2a.


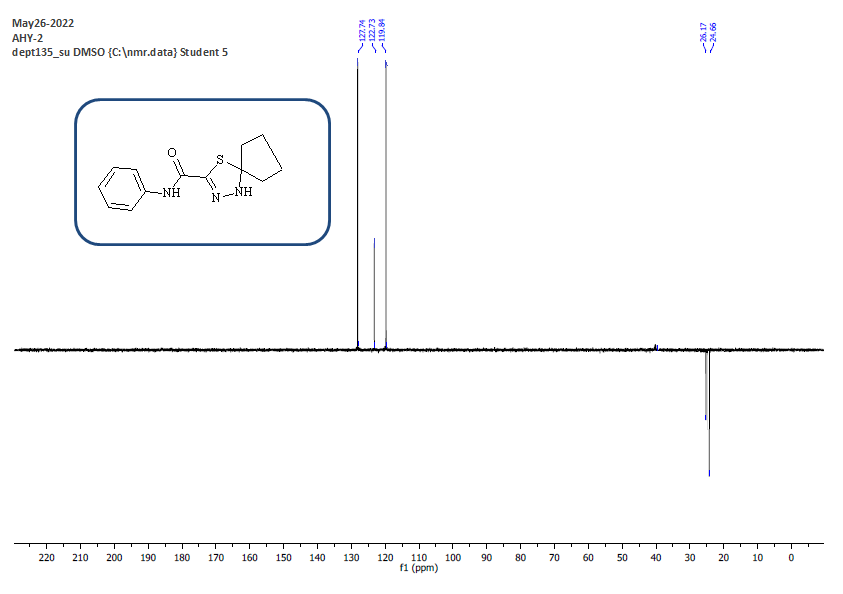


Figure of supplemental 5: IR of compound 3a.


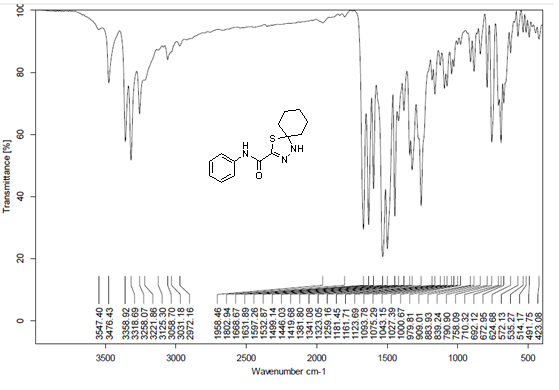


Figure of supplemental 6: ^1^HNMR spectrum of compound 3a.


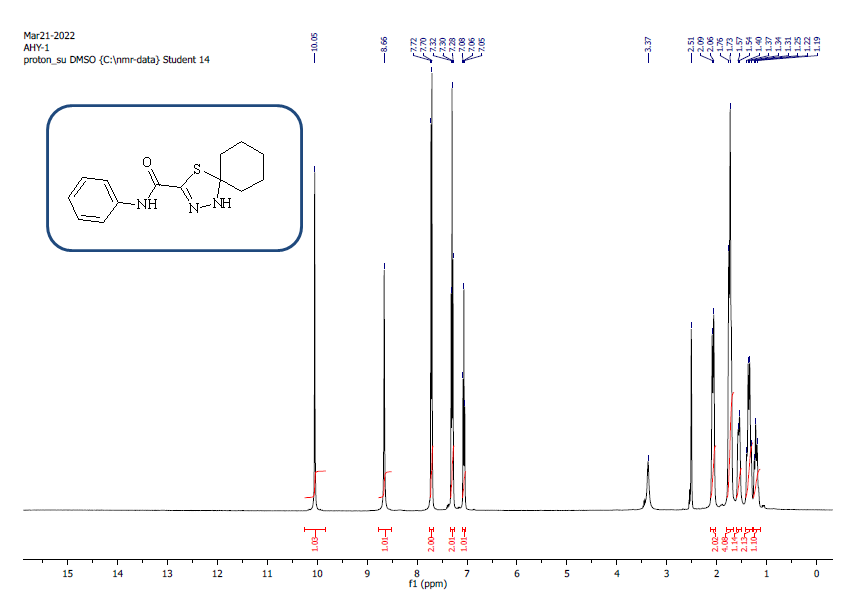


Figure of supplemental 7: ^13^CNMR of compound 3a.


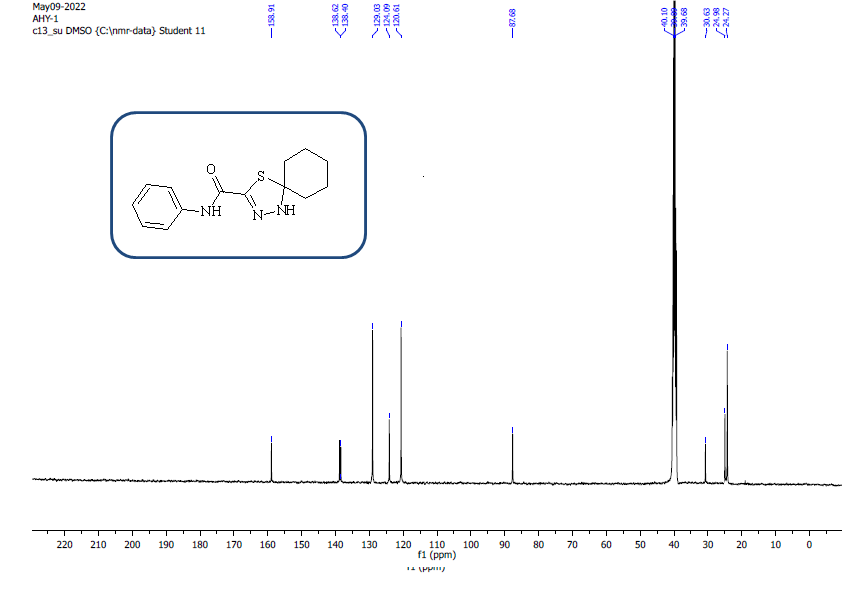


Figure of supplemental 8: Dept 135 of compound 3a.


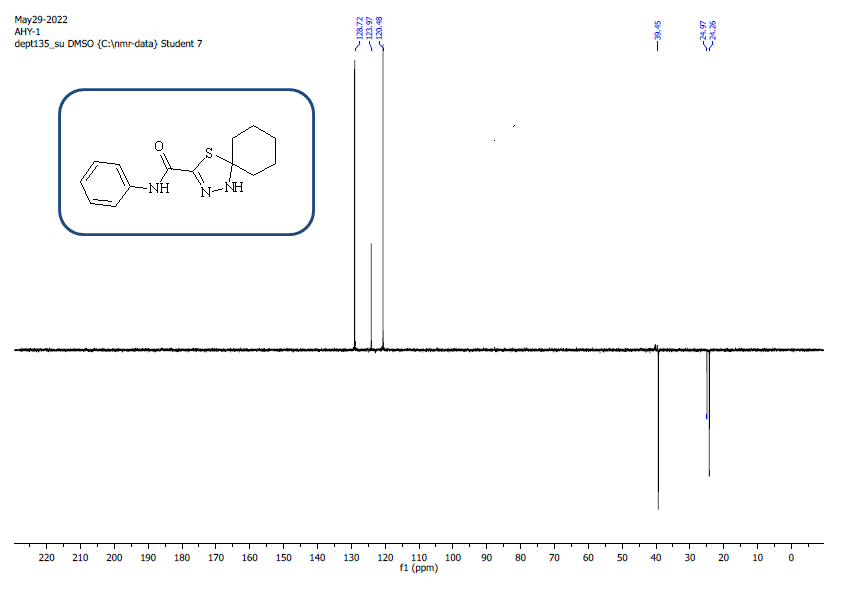


Figure of supplemental 9: IR of compound 4a.


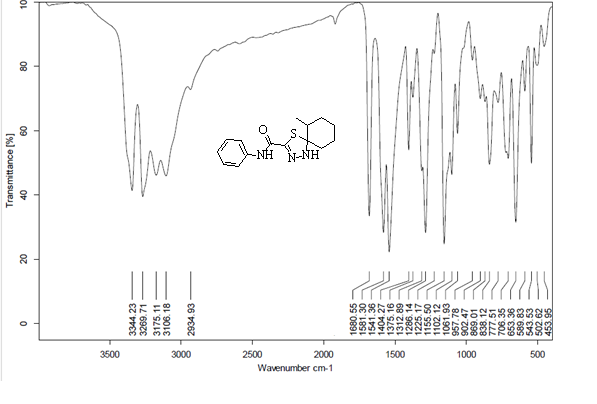


Figure of supplemental 10: ^1^HNMR of compound 4a.


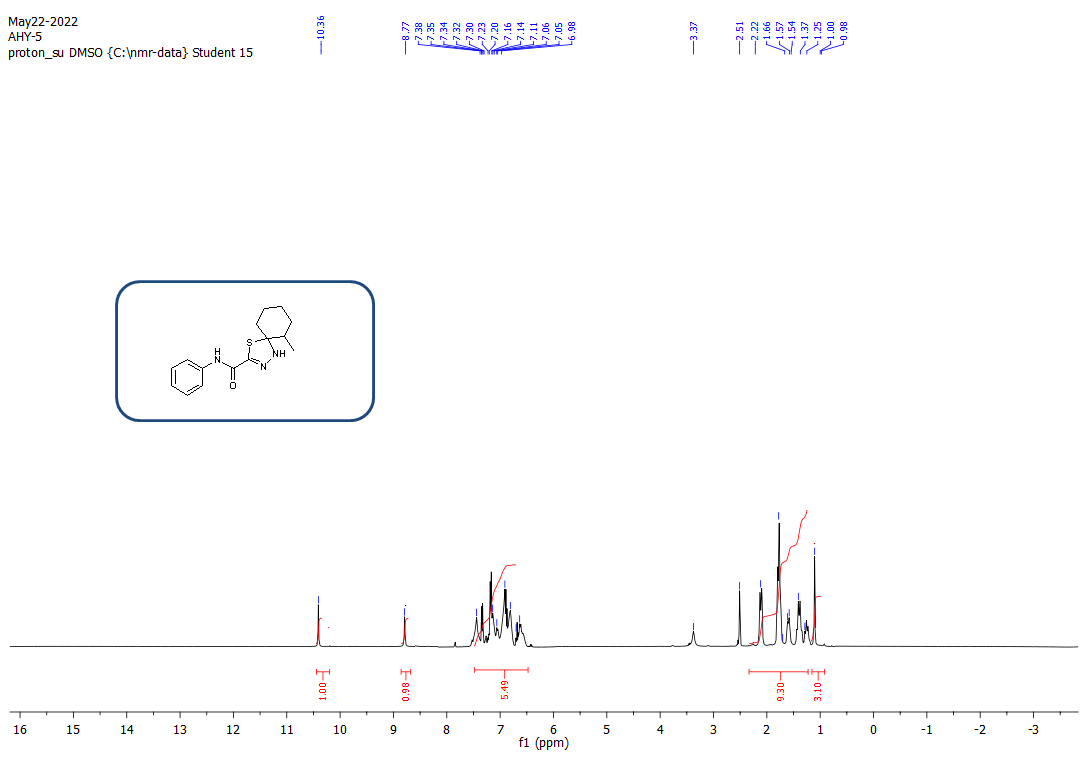


Figure of supplemental 11: IR of compound 5a.


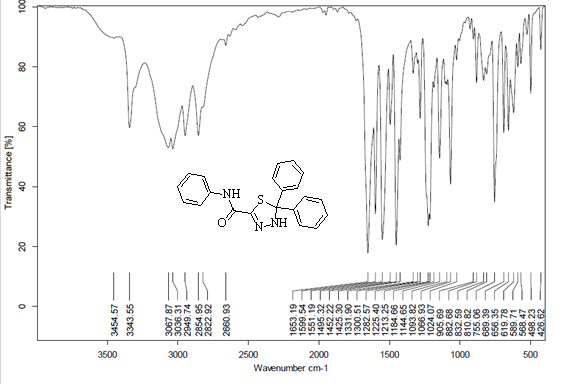


Figure of supplemental 12: ^1^HNMR of compound 5a.


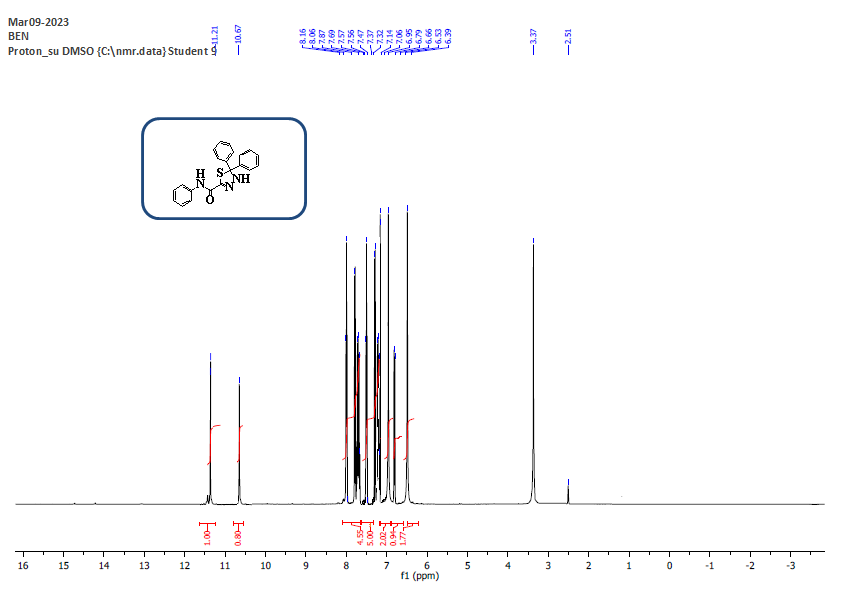


Figure of supplemental 13: ^13^C NMR of compound 5a.


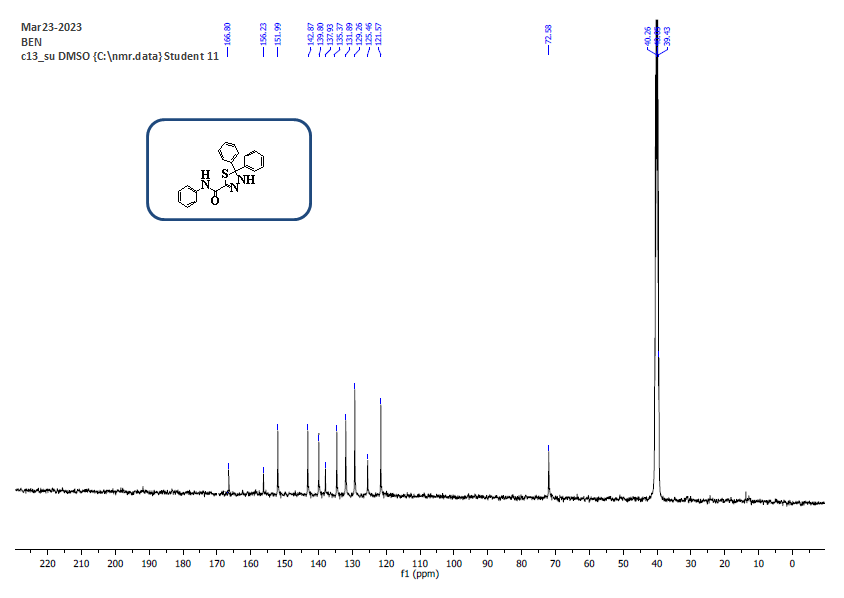


Figure of supplemental 14: IR of compound 6a.


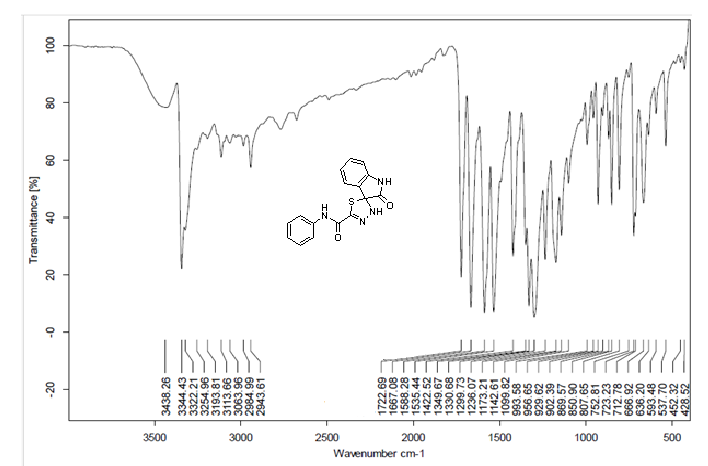


Figure of supplemental 15: ^1^HNMR of compound 6a.


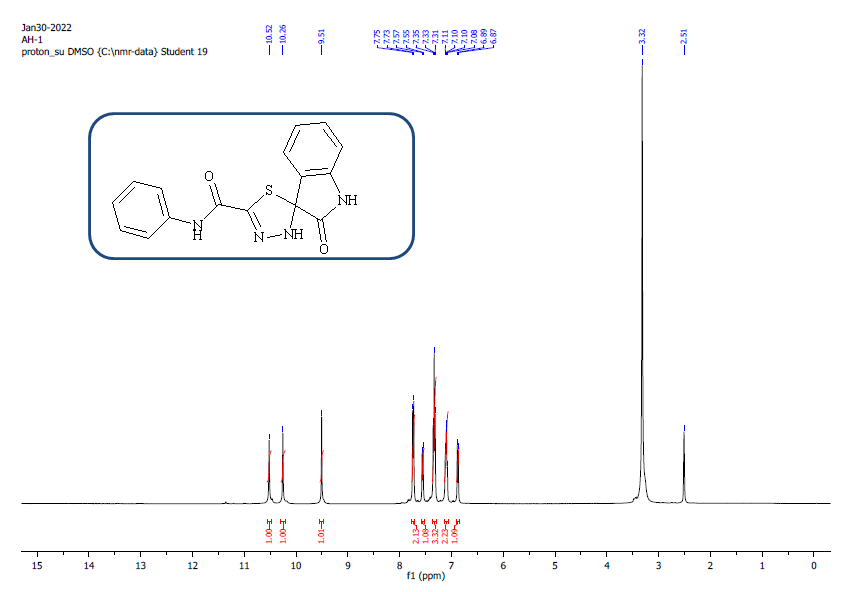


Figure of supplemental 16: D_2_O of compound 6a.


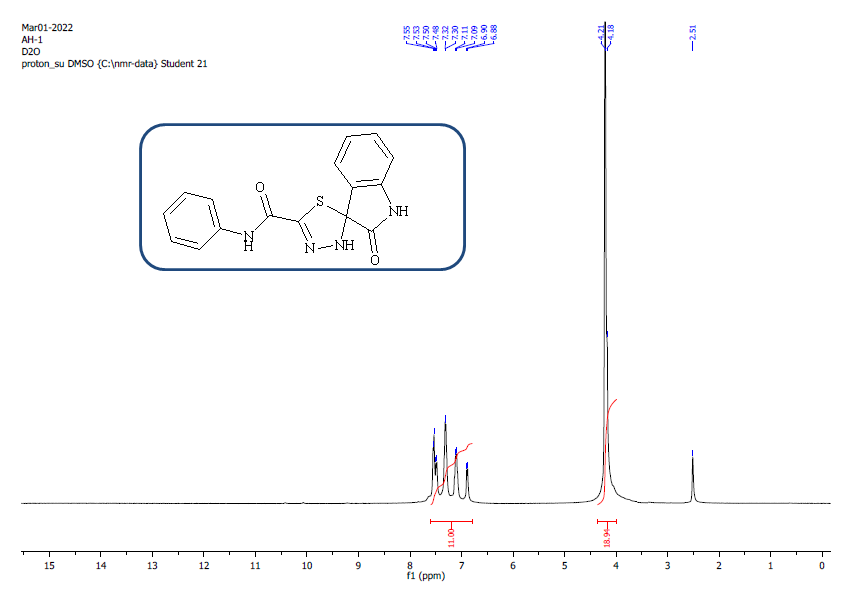


Figure of supplemental 17: IR of compound 6b.


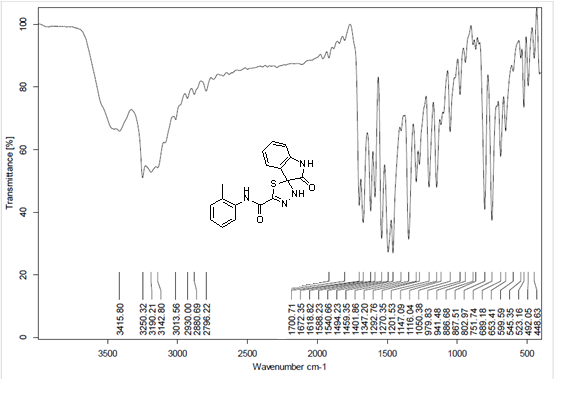


Figure of supplemental 18: ^1^H NMR of compound 6b.


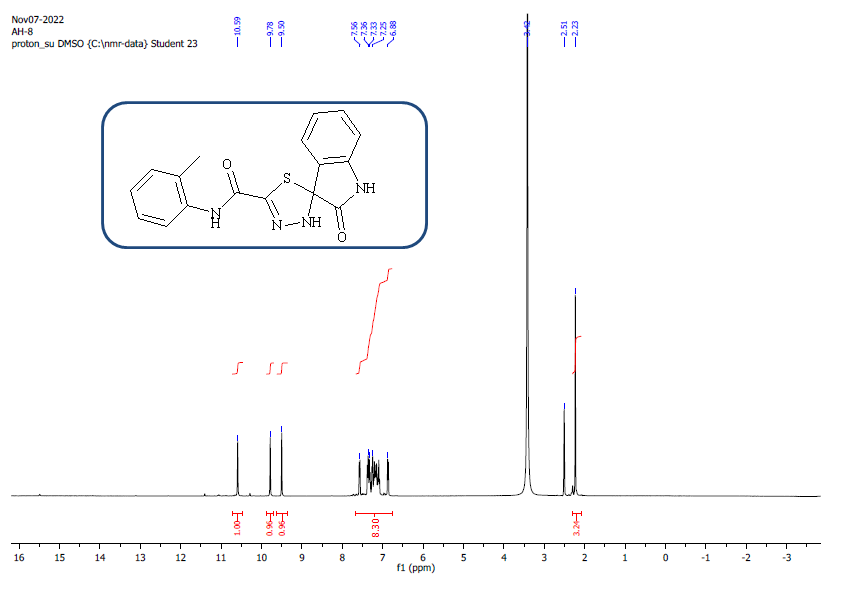


Figure of supplemental 19: ^13^C NMRof compound 6b.


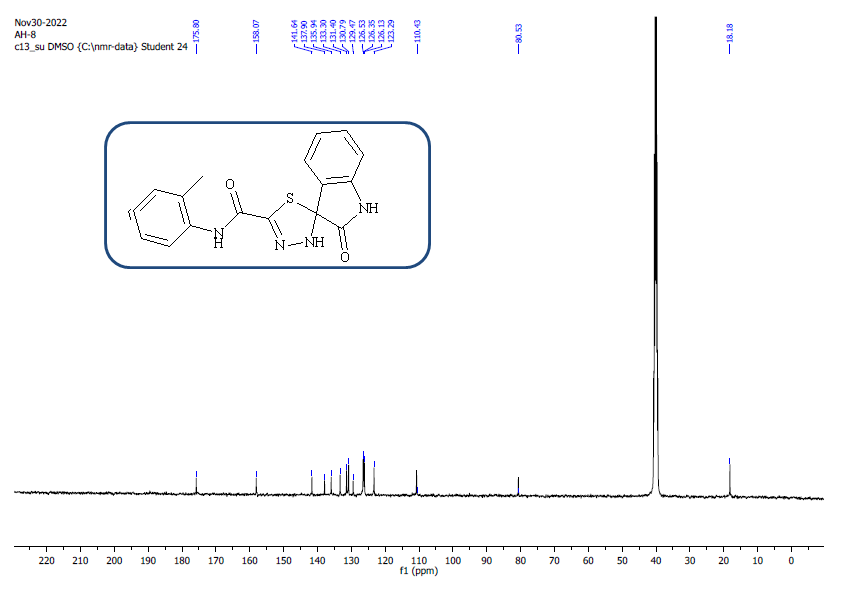


Figure of supplemental 20: IR of compound 6c.


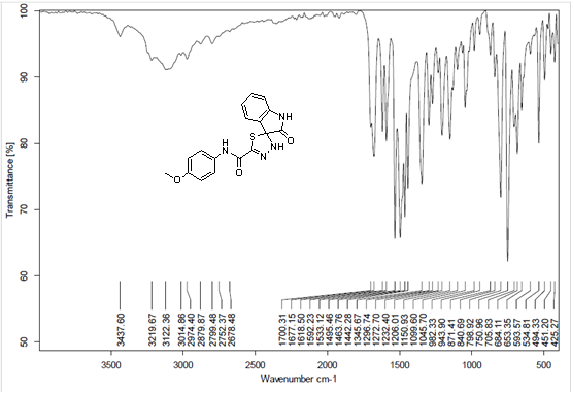


Figure of supplemental 21: ^1^H NMR of compound 6c.


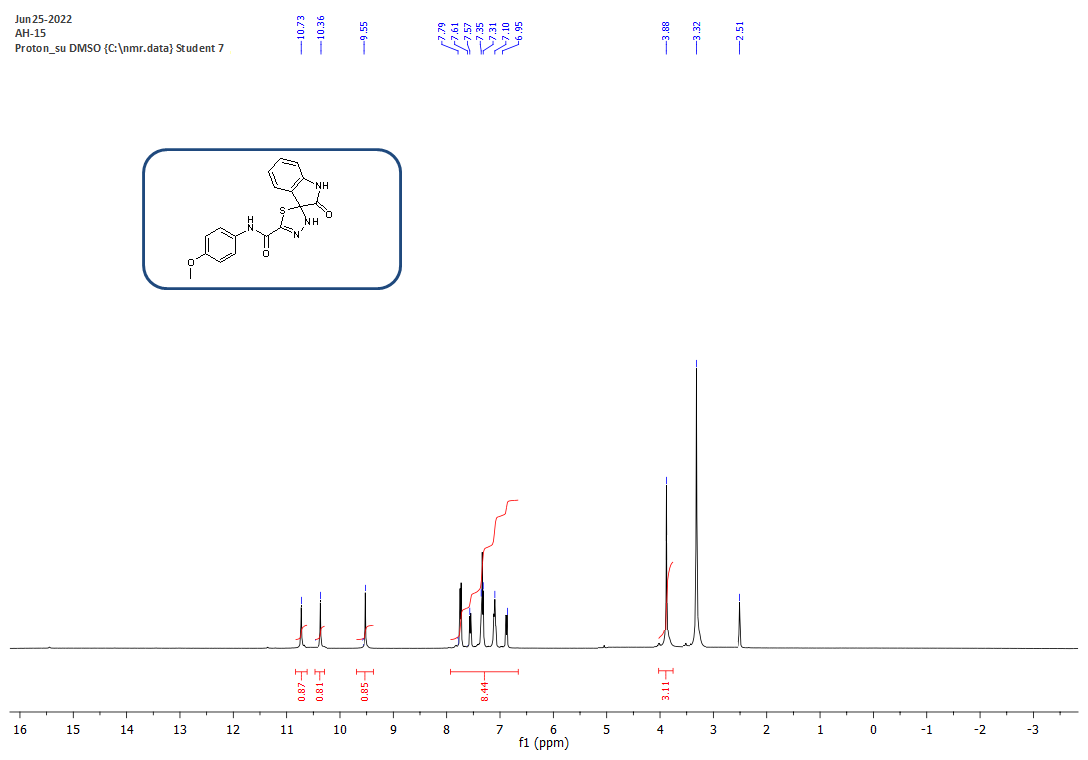


Figure of supplemental 22: IR of compound 6d.


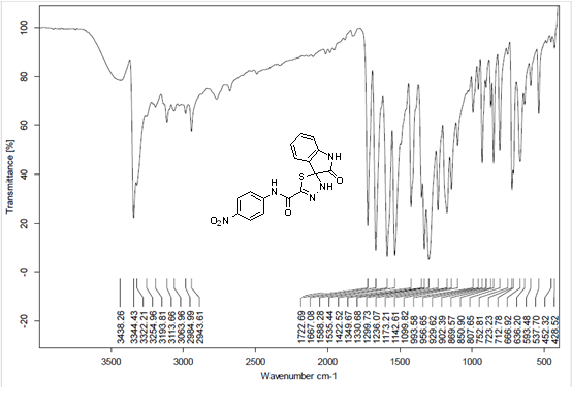


Figure of supplemental 23: ^1^H NMR of compound 6d.


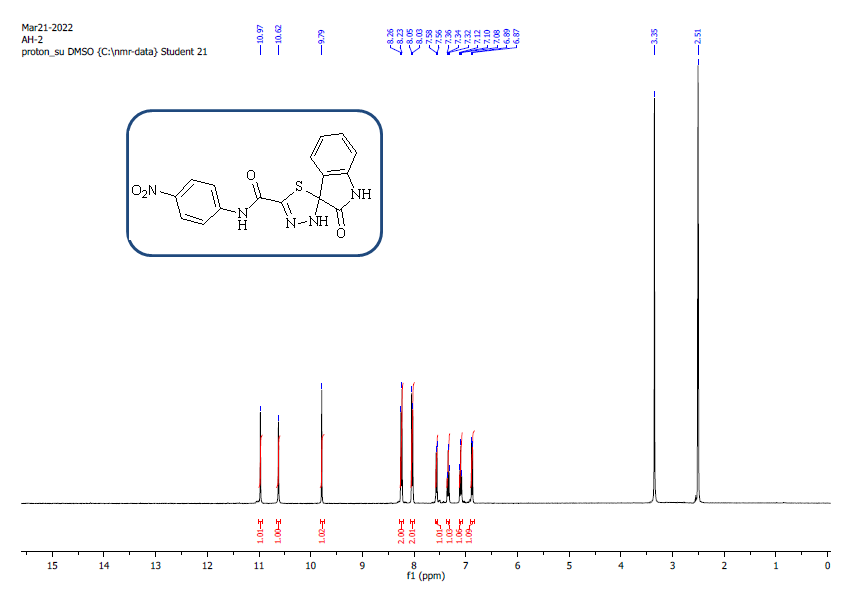


Figure of supplemental 24: D_2_O of compound 6d.


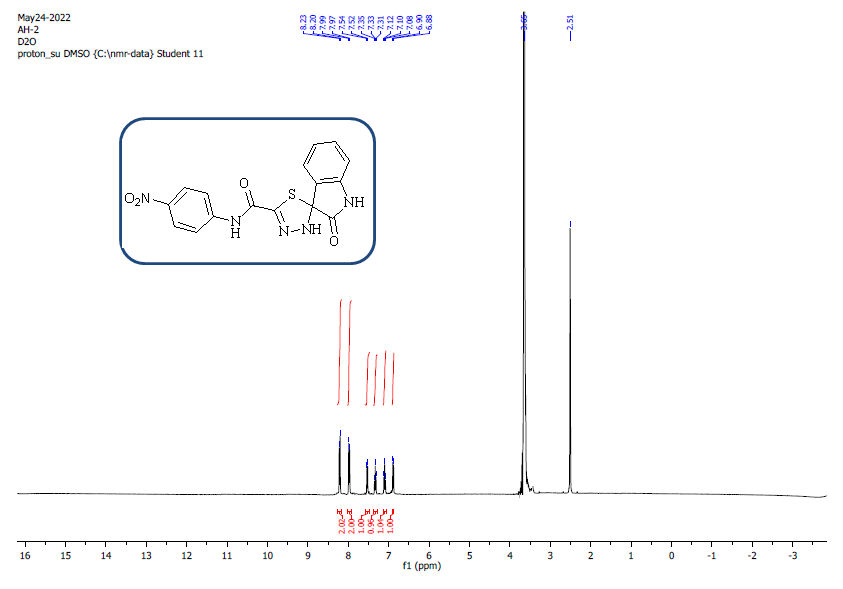


Figure of supplemental 25: ^13^C NMR of compound 6d.


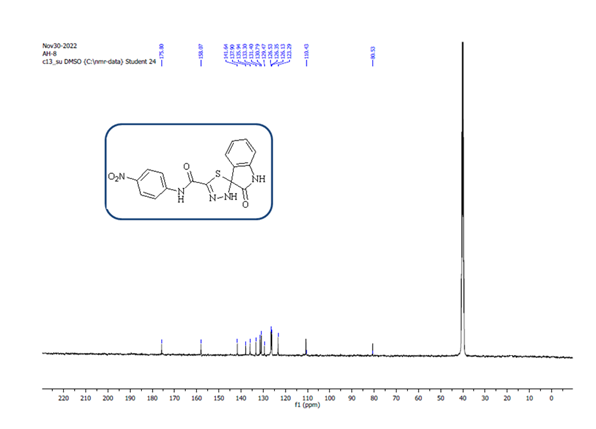


Figure of supplemental 26: IR of compound 6e.


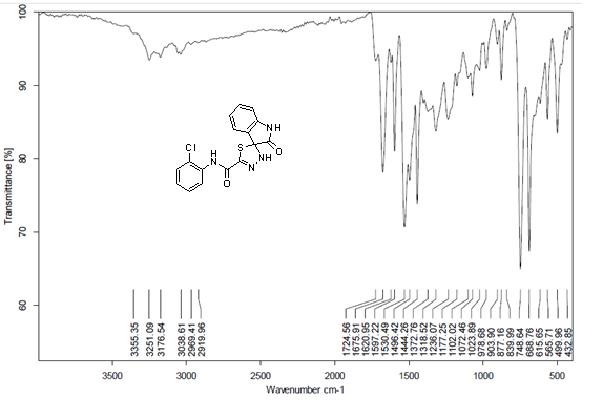


Figure of supplemental 27: ^1^H NMR of compound 6e.


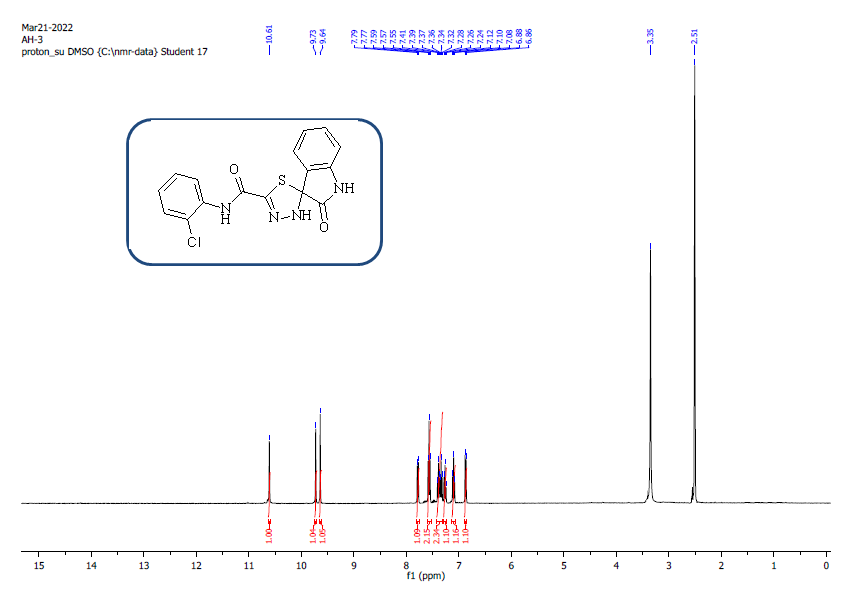


Figure of supplemental 28: ^1^H NMR of compound 6e.


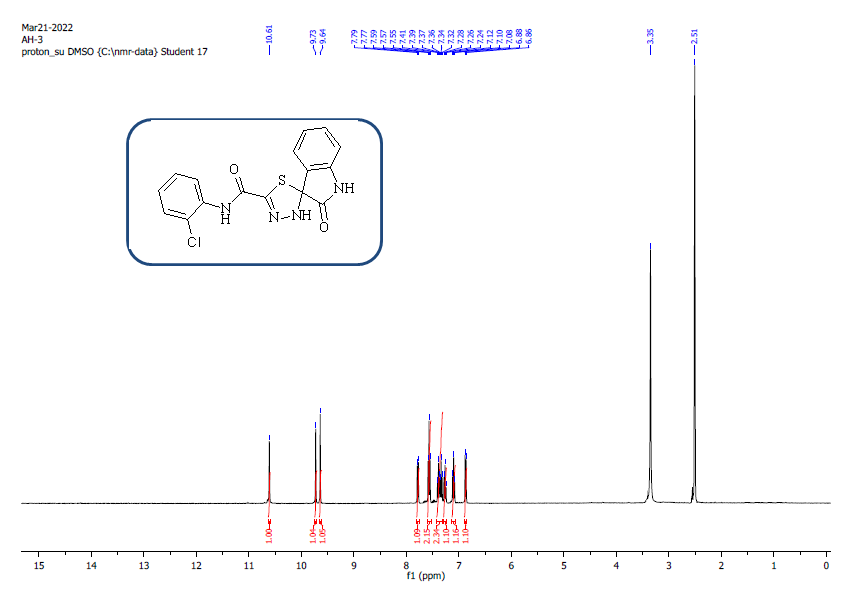


Figure of supplemental 29: D_2_O of compound 6e.


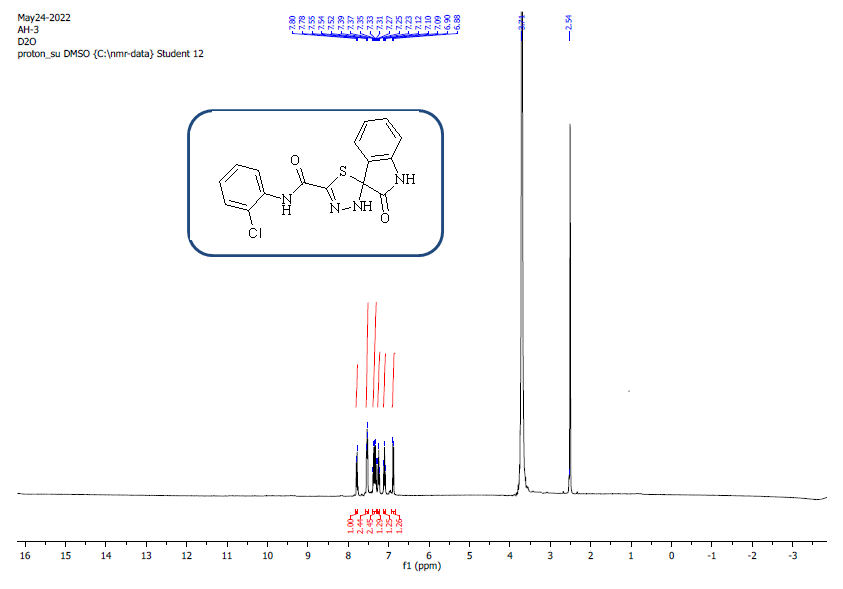


Figure of supplemental 30: 13C NMR of compound 6e.


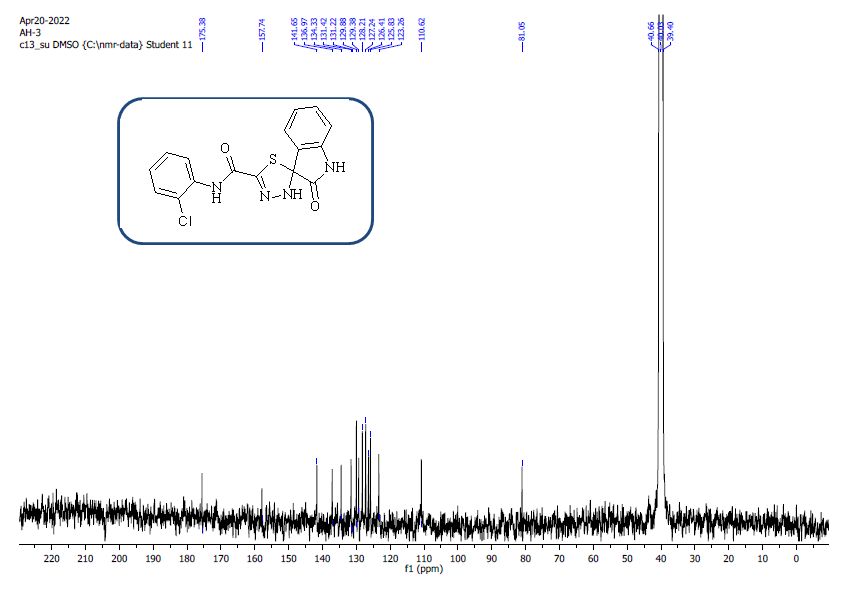


Figure of supplemental 31: IR of compound 7a.


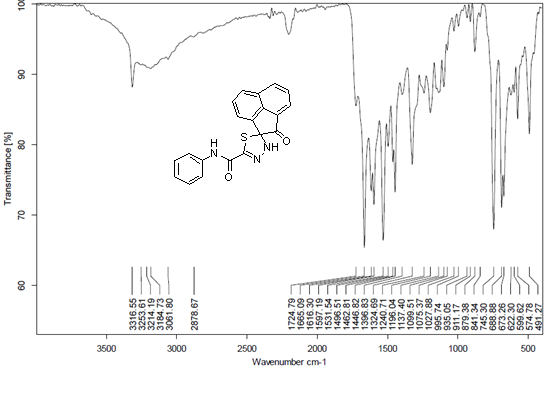


Figure of supplemental 32: ^1^H NMR of compound 7a.


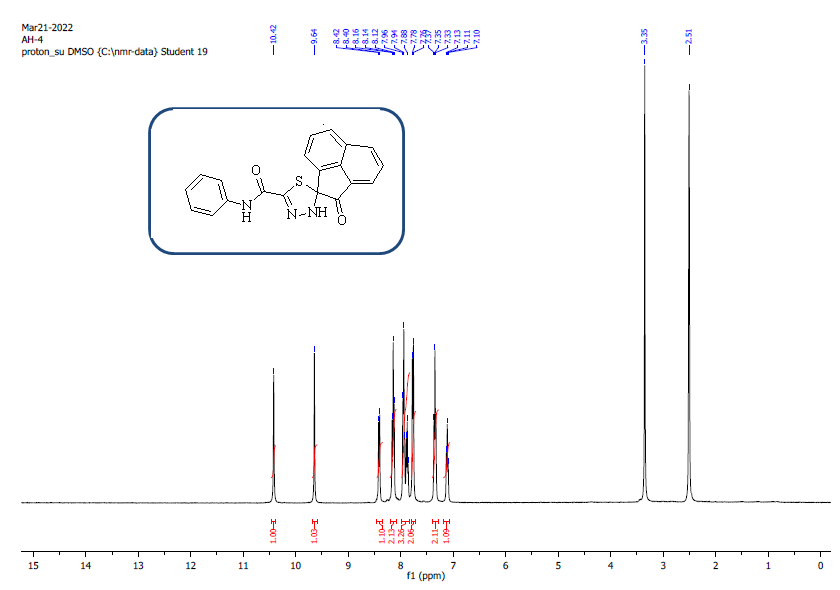


Figure of supplemental 33: D_2_O of compound 7a.


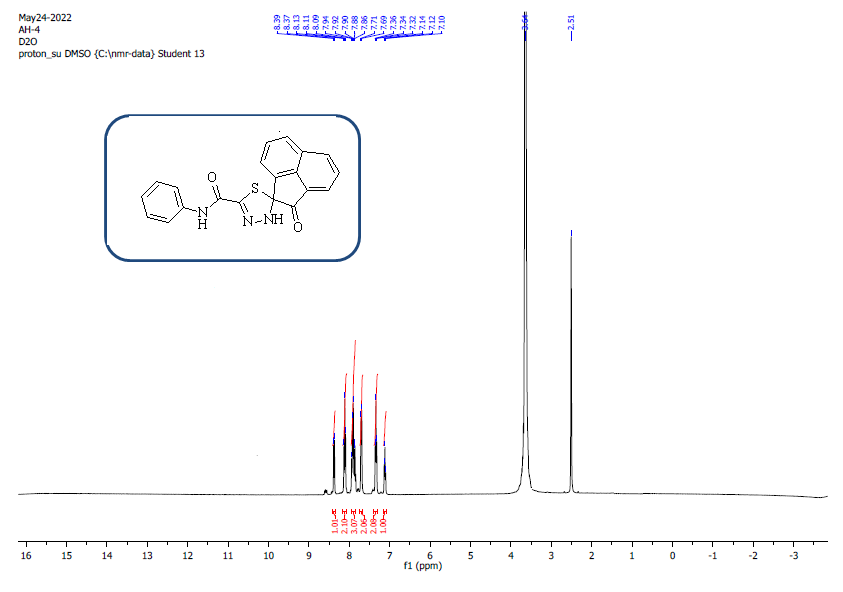


Figure of supplemental 34: ^1^H NMR of compound 2.


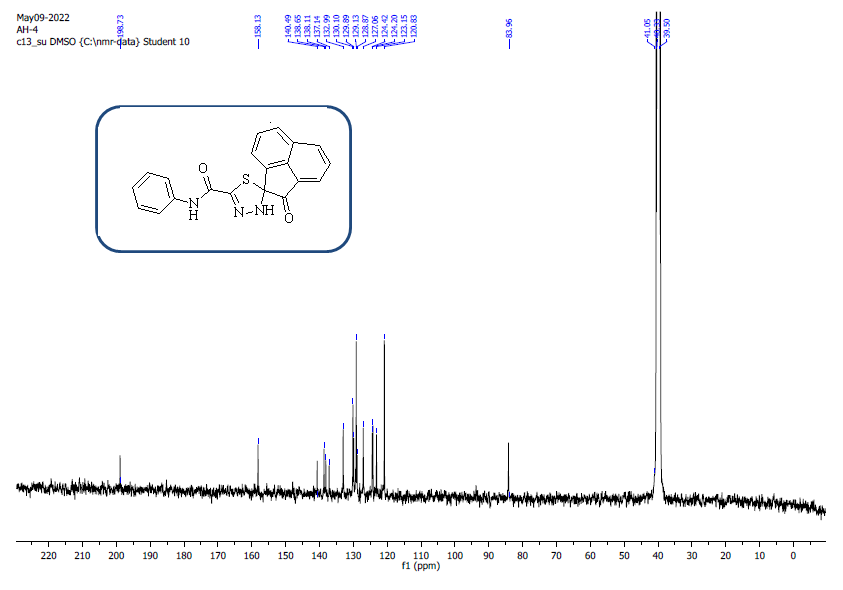


Figure of supplemental 35: IR of compound 7c.


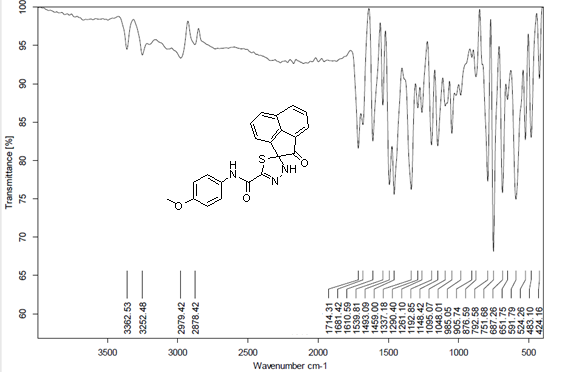


Figure of supplemental 36: ^1^H NMR of compound 7c.


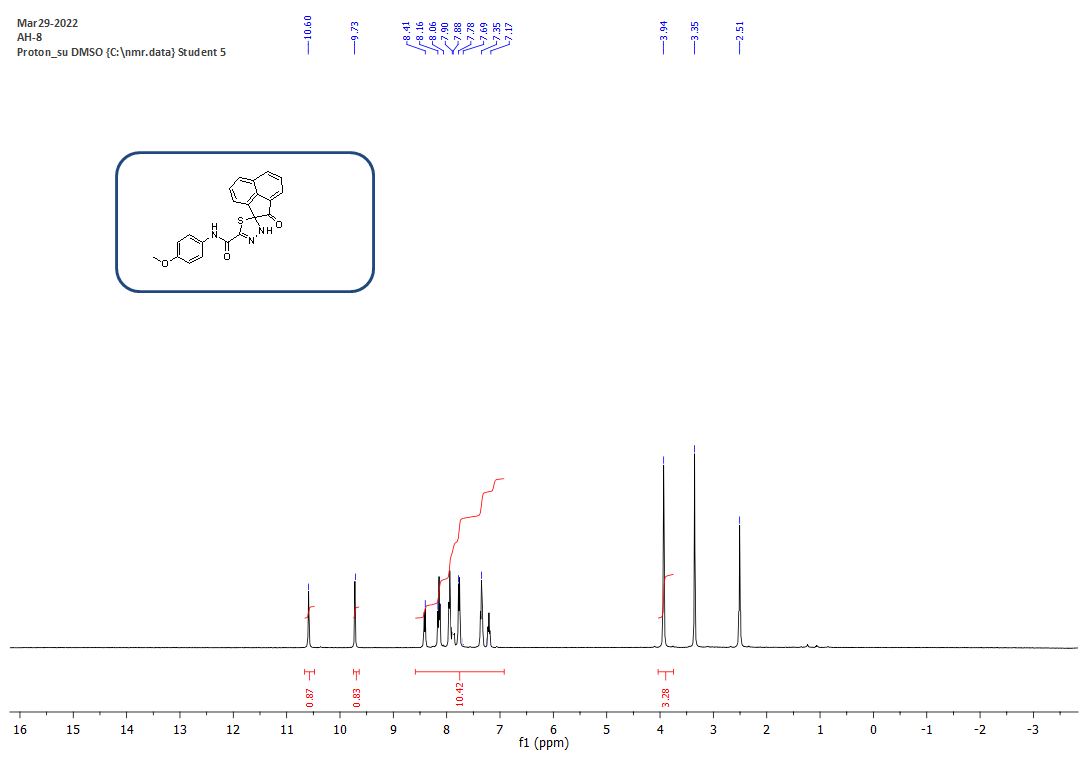


Figure of supplemental 37: IR of compound 8a.


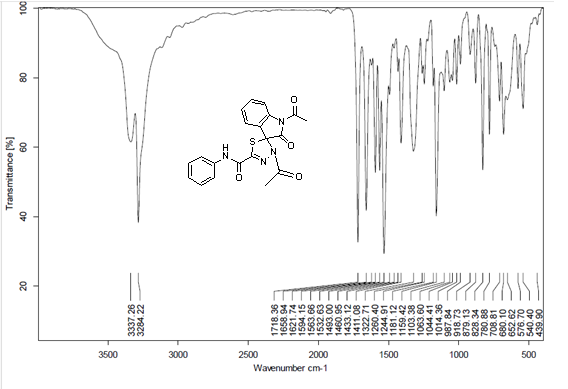


Figure of supplemental 38: ^1^H NMR of compound 8a.


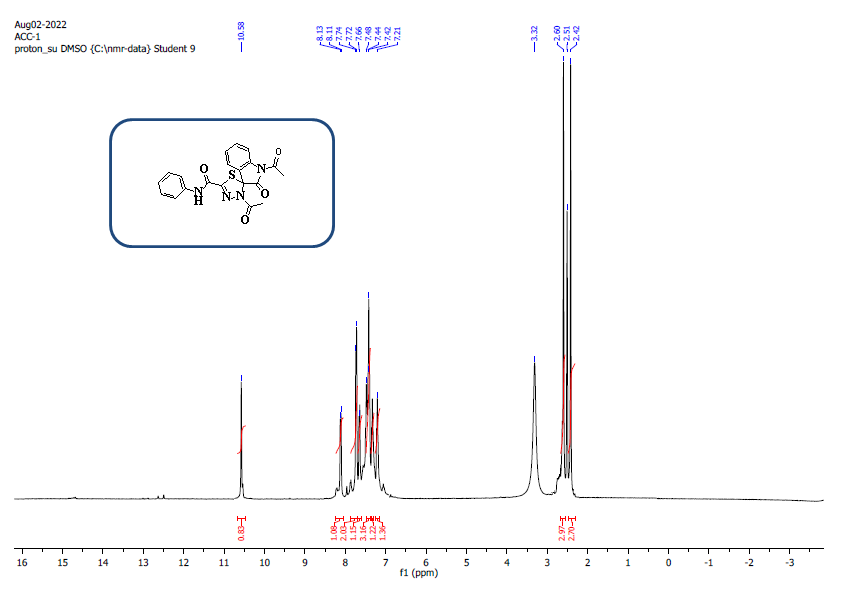


Figure of supplemental 39: ^13^C NMR of compound 8a.


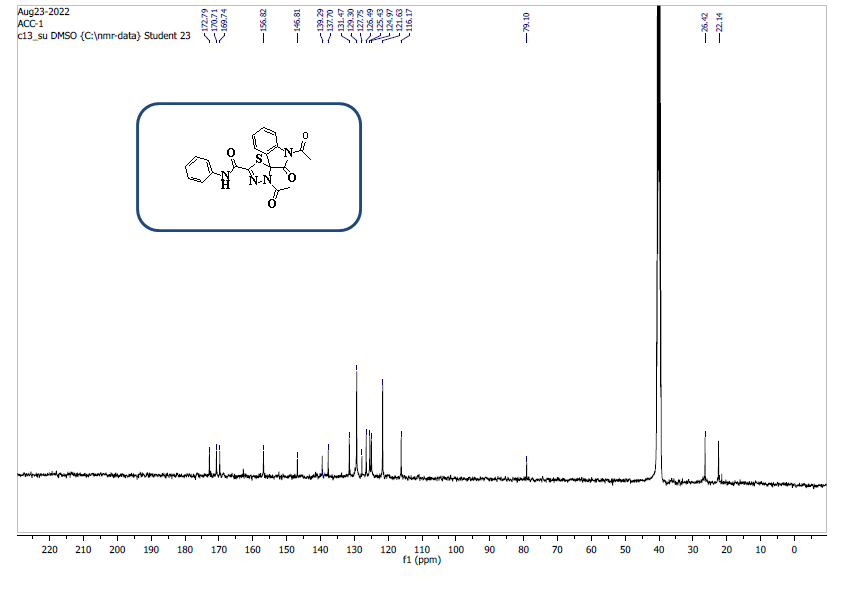


Figure of supplemental 40: IR of compound 9a.


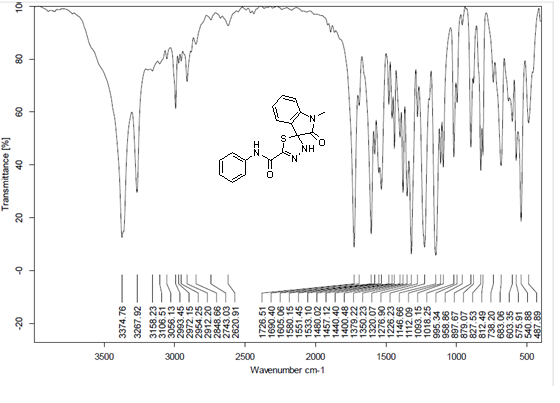


Figure of supplemental 41: ^1^H NMR of compound 9a.


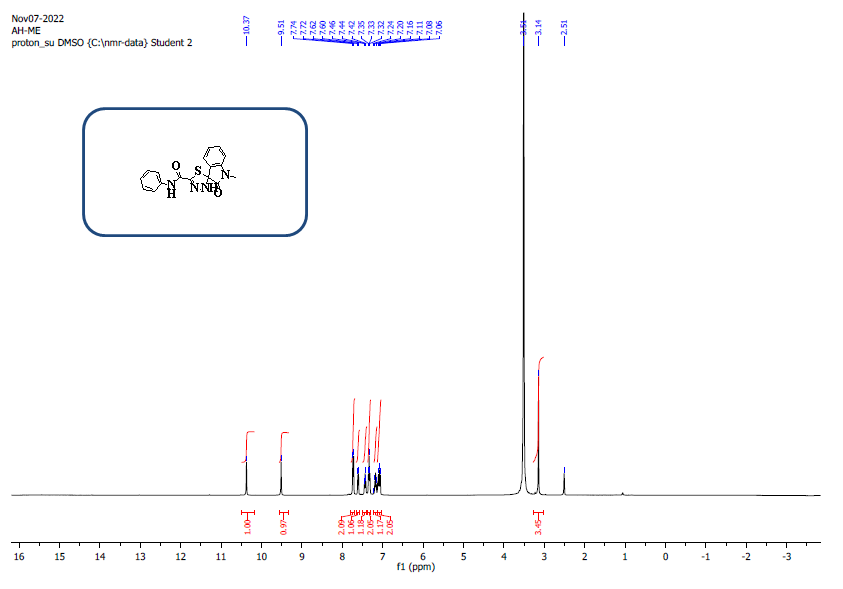


Figure of supplemental 42: ^13^C NMR of compound 9a.


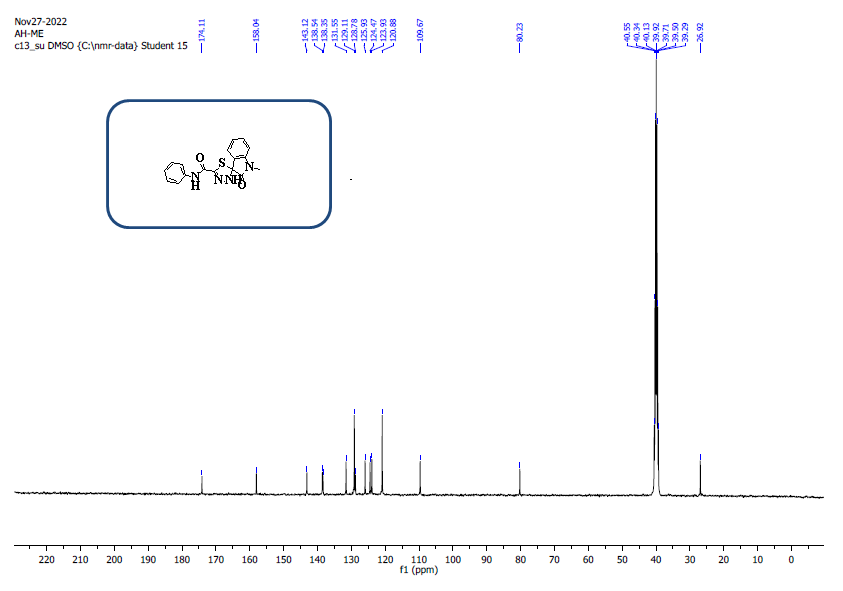


Figure of supplemental 43: IR of compound 10a.


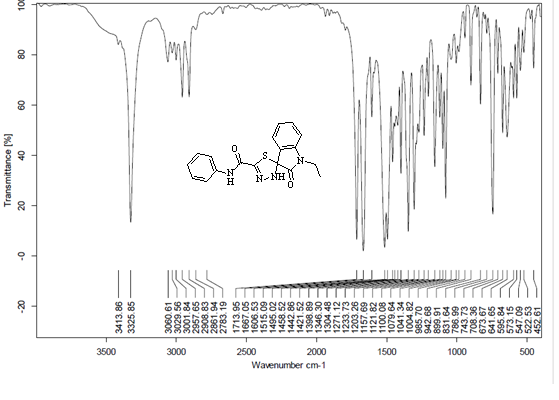


Figure of supplemental 44: ^1^H NMR of compound 10a.


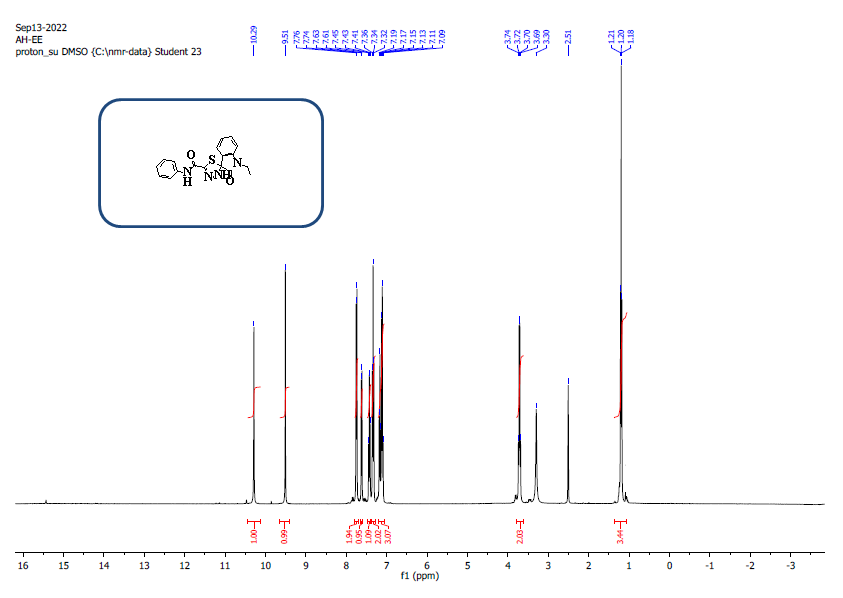


Figure of supplemental 45: ^13^C NMR of compound 10a.


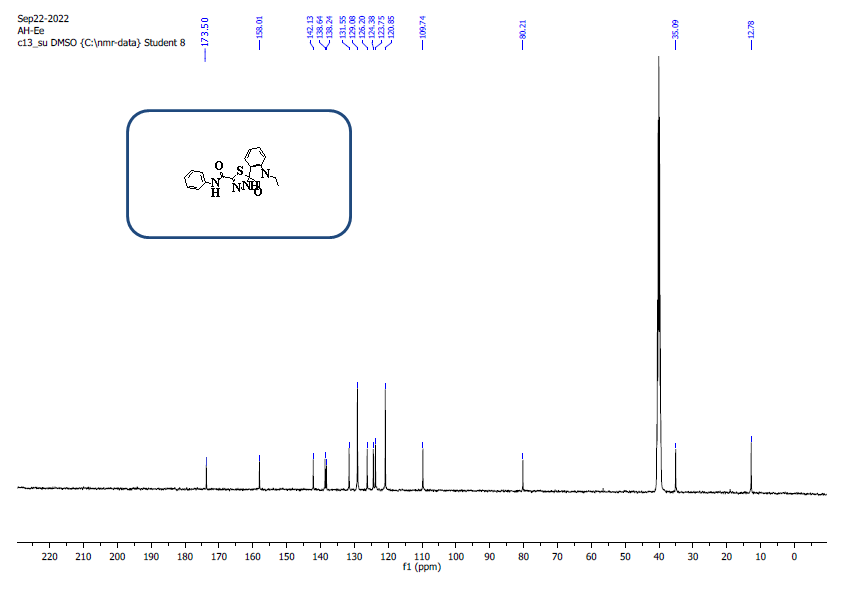


Figure of supplemental 46: Dept of compound 10a.


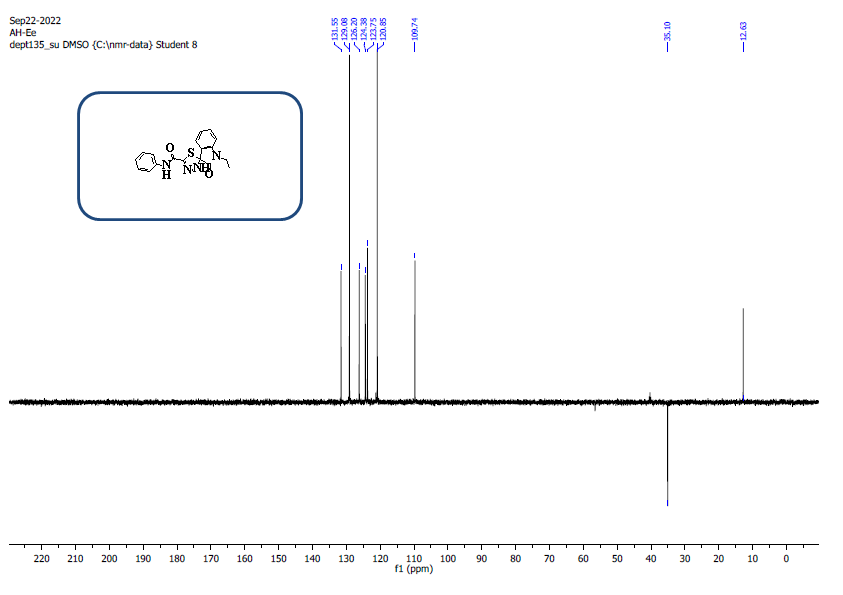


Figure of supplemental 47: IR of compound 11a.


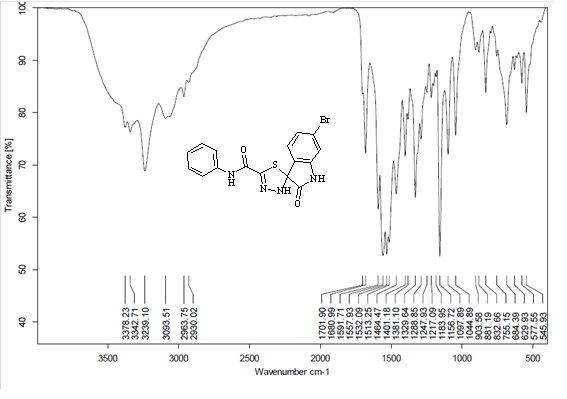


Figure of supplemental 48: ^1^H NMR of compound 11a.


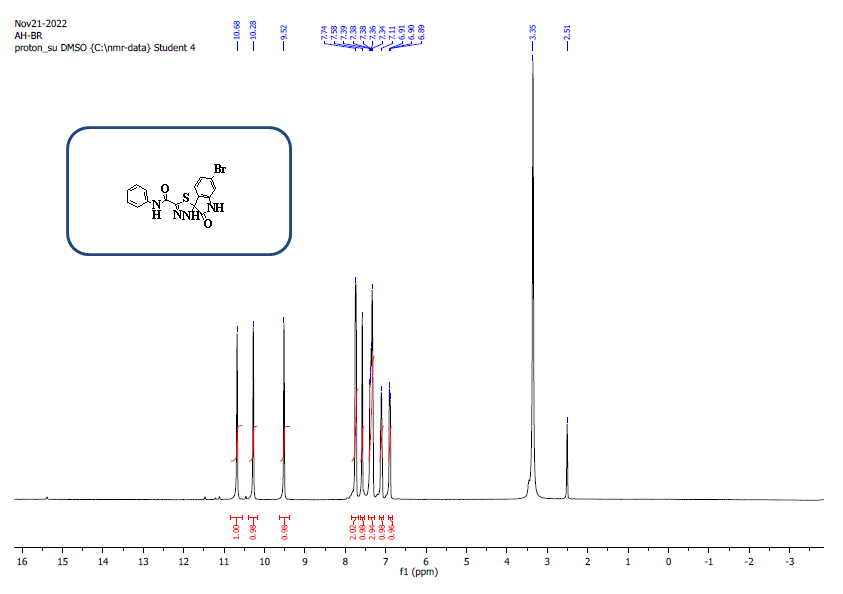


Figure of supplemental 49: ^13^C NMR of compound 11a.


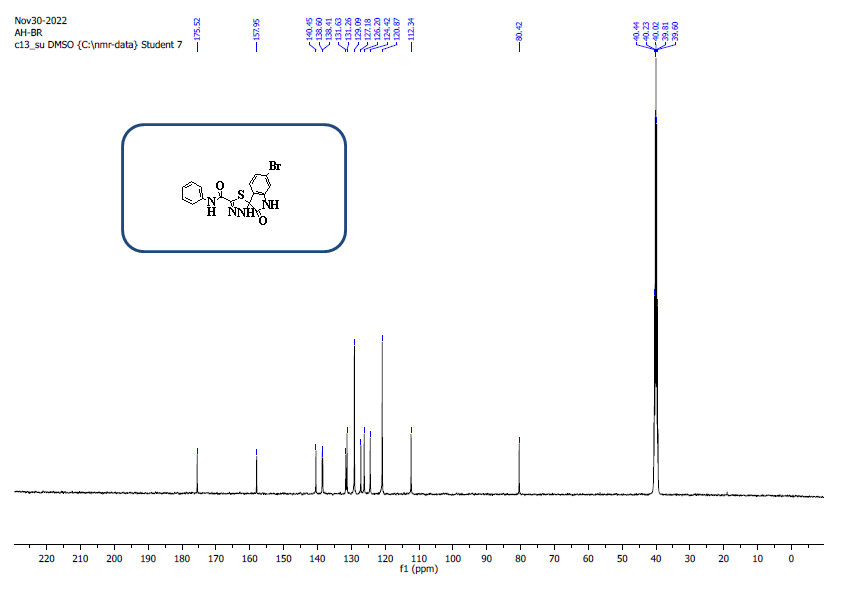


Figure of supplemental 50: IR of compound 12a.


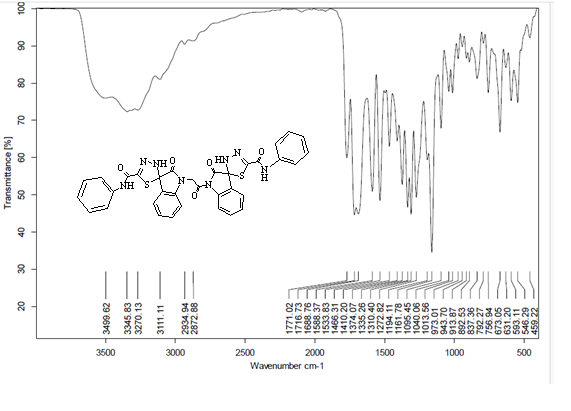


Figure of supplemental 51: ^1^H NMR of compound 12a.


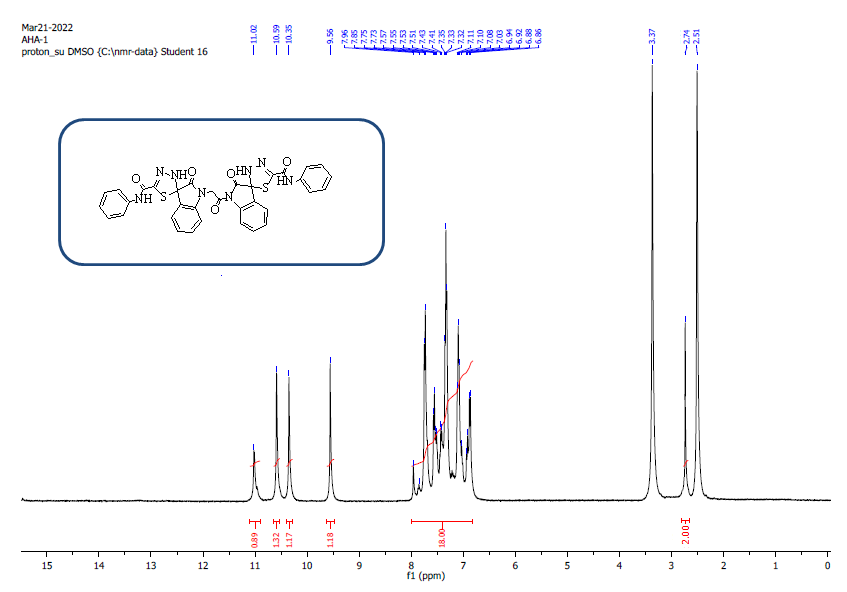


Figure of supplemental 52: D_2_O of compound 12a.


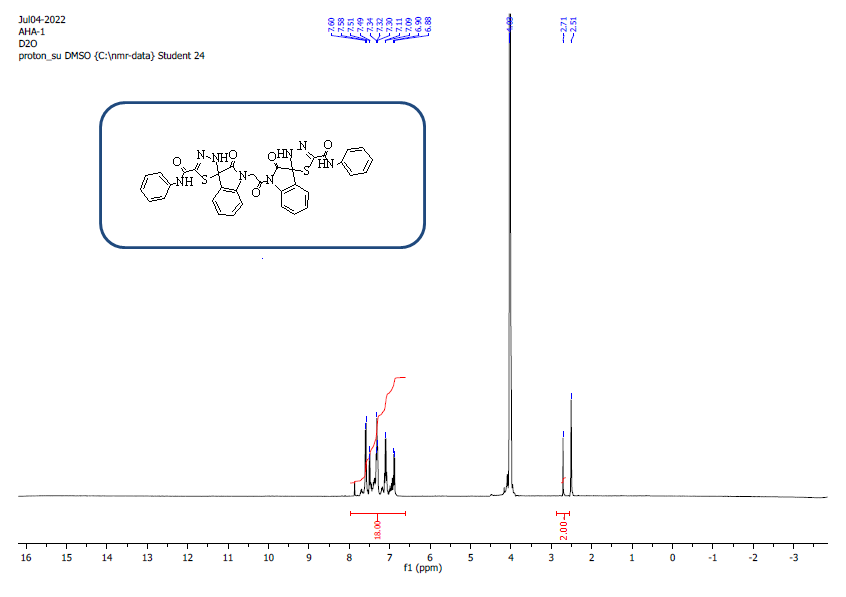


Figure of supplemental 53: ^13^C NMR of compound 12a.


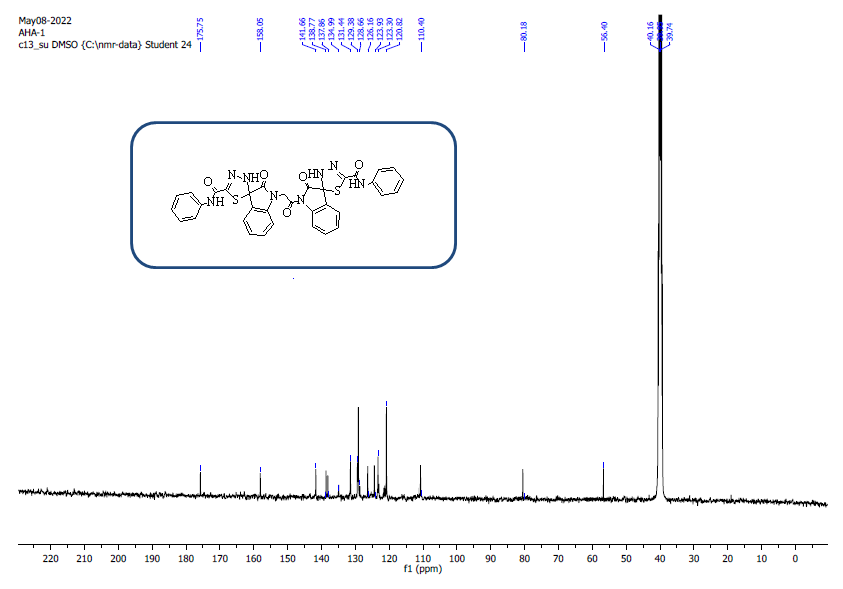


Figure of supplemental 54: Dept of compound 12a.


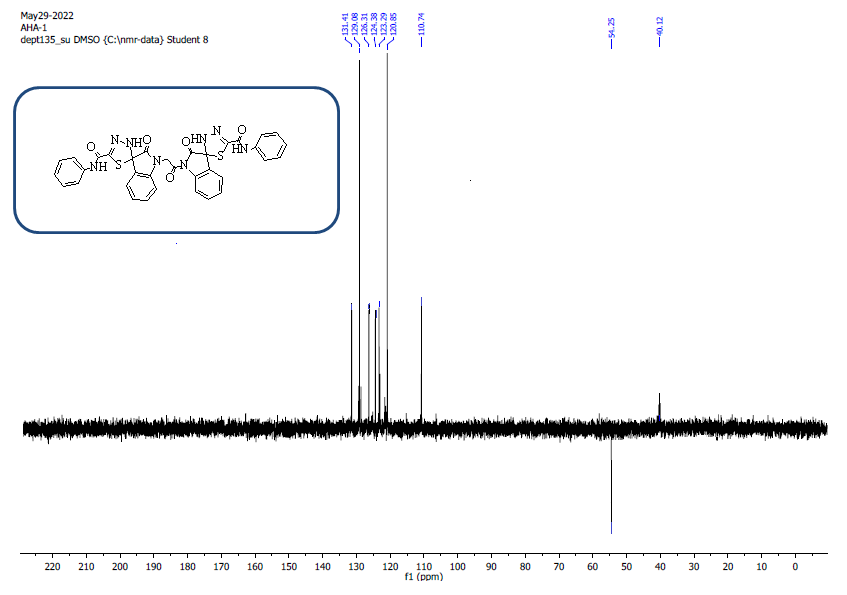


**Supplementary Information**

**S.I. Docking validation**

The re-docking and superimposition approach was used in order to verify the validity of the docking operation [1-3]. The native ligand that was associated with the 5KIR enzyme was removed and then re-docked into the active site. The standard procedure for docking was not altered in any way throughout the operation. This was done in order to guarantee that the inhibitor binds precisely to the active site cleft and must exhibit less variation when compared to the real co-crystallized complex. After that, the re-docked complex was overlaid with the original co-crystallized ligand, and the root mean square deviation (RMSD) was computed. These were carried out to verify the docking method in order to guarantee that docking would be successful.

**List of Figures**

1. Fig. (S55): 3D representation of the superimposition of the co-crystallized (green) and the re-docking pose (pink) of the ligand in 5KIR binding site.

| 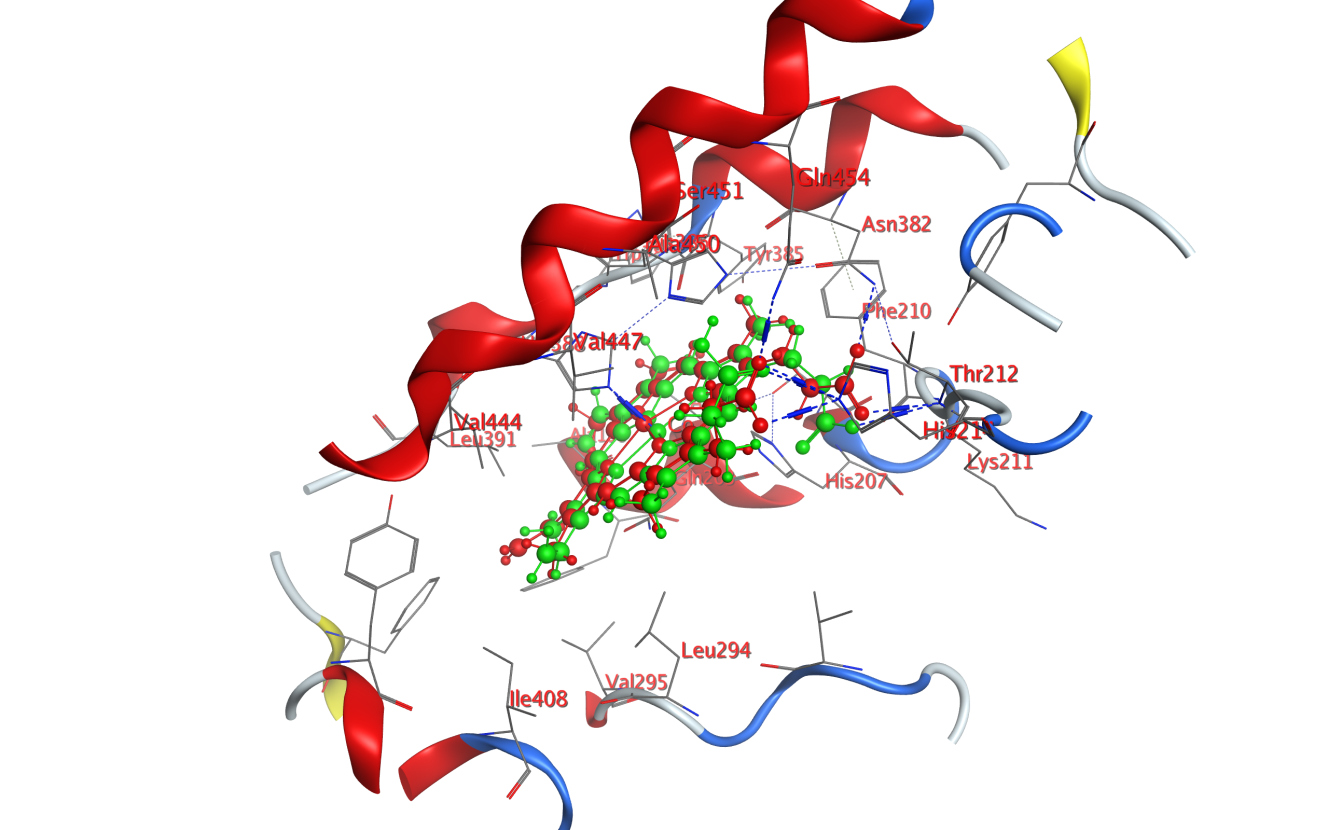 |
| --- |
| Fig. (S55): 3D representation of the superimposition of the co-crystallized (red) and the re-docking pose (green) of the ligand in 5IKT binding site. |

[1] C. Shivanika, D. Kumar, V. Ragunathan, P. Tiwari, A. Sumitha, Molecular docking, validation, dynamics simulations, and pharmacokinetic prediction of natural compounds against the SARS-CoV-2 main-protease, Journal of biomolecular structure & dynamics (2020) 1.

[2] E.M. Terefe, A. Ghosh, Molecular Docking, Validation, Dynamics Simulations, and Pharmacokinetic Prediction of Phytochemicals Isolated from Croton dichogamus Against the HIV-1 Reverse Transcriptase, Bioinformatics and Biology Insights 16 (2022) 11779322221125605.

[3] K.E. Hevener, W. Zhao, D.M. Ball, K. Babaoglu, J. Qi, S.W. White, R.E. Lee, Validation of molecular docking programs for virtual screening against dihydropteroate synthase, Journal of chemical information and modeling 49(2) (2009) 444-460.
